# Dual-Loop Resilient Scheduling with Predictive Path Reconfiguration for Deterministic Industrial Wireless Networks

**DOI:** 10.3390/s26206554

**Published:** 2026-10-15

**Authors:** Kuo-Feng Huang

**Affiliations:** Department of Information Technology, Ling Tung University, No. 1, Lingdong Rd., Nantun Dist., Taichung City 408284, Taiwan; sailerhuang@teamail.ltu.edu.tw

**Keywords:** IEEE 802.15.4-TSCH, Software-Defined Networking, resilient scheduling, deterministic industrial communication, narrowband-interference mitigation

## Abstract

**Highlights:**

**What are the main findings?**

- Formulates resilient 6TiSCH scheduling as an explicit mixed-integer linear program (MILP), proves its NP-completeness, and introduces the Dual-Loop Intelligent Resilient Scheduling (DL-IRS) architecture with the Conflict-Aware Spatial-Spectral Allocation (CASA) heuristic.
- Achieves a 96.8% Packet Delivery Ratio (PDR) under severe narrowband interference (SNR = 0 dB), representing an absolute improvement of 35.3 percentage points over standard 6TiSCH scheduling (61.5%).

**What are the implications of the main findings?**

- Decouples transient channel degradation from persistent path failures into distinct inner and outer resilience loops, providing low-disruption, deterministic communication for Industry 4.0 applications.
- Achieves significant spatial-reuse efficiency (a +147.6% relative SSRE gain; 0.163 versus 0.066 for standard 6TiSCH) and bounded signaling overhead (under 5.1%), enabling sustainable network lifetimes up to 168 days for battery-operated industrial sensor nodes.

**Abstract:**

To meet the deterministic and low-latency communication demands of Industry 4.0, this paper formulates resilient scheduling in interference-prone 6TiSCH industrial wireless networks as an explicit mixed-integer linear program (MILP) and proposes Dual-Loop Intelligent Resilient Scheduling (DL-IRS), a Software-Defined Networking (SDN) architecture. An inner loop performs minimum-perturbation, local channel-offset remapping to absorb transient narrowband interference; an outer loop performs make-before-break activation of a shadow schedule, triggered by trend-based early warning when local recovery is insufficient; shadow resources are reserved in advance and the schedule update is installed before an explicit activation slot. Both loops draw on a Conflict-Aware Spatial-Spectral Allocation (CASA) heuristic, whose underlying decision problem is proven NP-complete via a reduction from graph coloring. Cooja/Contiki-NG simulations demonstrate that under an SNR = 0 dB narrowband-interference condition, DL-IRS sustains a packet delivery ratio (PDR) of 96.8%, representing a 35.3 percentage point improvement over standard 6TiSCH scheduling. The detection-to-activation chain of the outer loop is reported explicitly (38.0 ms in a relay-failure experiment) rather than assumed to be zero. This chain was measured for the compute-on-trigger branch only; the Phase A proactive reservation mechanism is specified as part of the architecture but is not evaluated experimentally in this paper.

## 1. Introduction

With the advent of the Industry 4.0 paradigm, the Industrial Internet of Things (IIoT) has emerged as a fundamental cornerstone for realizing smart manufacturing. In the sophisticated environments of automated factories, thousands of sensors and actuators necessitate high-frequency data exchange over wireless networks. To meet the stringent deterministic-transmission requirements of such mission-critical applications, the IEEE 802.15.4-TSCH (Time-Slotted Channel Hopping) protocol [1,2] was developed. By integrating Time Division Multiple Access (TDMA) with channel-hopping mechanisms, this protocol provides a low-power communication framework with robust interference immunity. However, in practical deployments, the ubiquity of narrowband interference (NBI) and electromagnetic interference (EMI) generated by heavy machinery often renders standard 6TiSCH scheduling mechanisms inadequate, making it challenging to maintain a stable packet delivery ratio (PDR) within dynamic industrial landscapes.

Contemporary industrial wireless networks face a critical trade-off between reliability and resource efficiency. Conventional scheduling algorithms predominantly employ reactive mechanisms, in which resource reallocation is triggered only after a link failure has been detected. This delayed response not only incurs significant end-to-end latency but may also interrupt closed-loop automated control. The common alternative of over-provisioning redundant paths to enhance reliability frequently wastes spectral resources and increases node energy consumption. Developing a scheduling architecture that proactively senses environmental fluctuations and reconfigures resources with a scoped, measurable disruption budget therefore remains a pivotal technical challenge.

This work addresses resilient scheduling in interference-prone 6TiSCH networks, where short-lived channel degradation and persistent topology-level disruptions require different recovery scopes. Unlike reactive schedulers that rebuild resources only after failure detection, and unlike proactive schedulers that adapt only a channel-selection policy, DL-IRS couples (i) a local, minimum-perturbation channel-remapping loop with (ii) a predictive, make-before-break path-reconfiguration loop. The two loops share a common conflict-aware resource model but act on distinct decision variables and distinct timescales; Table 1 makes this distinction explicit and falsifiable so that it can be verified rather than asserted.

Technical challenges. Three challenges motivate the design. *(C1) Timescale and scope mismatch:* transient narrowband interference calls for a fast, low-perturbation reaction on a single link, whereas node failures call for structural, end-to-end changes; applying one recovery action to both either over-reacts to transient events, inflating control overhead, or under-reacts to persistent ones, prolonging outages. *(C2) Auditability of the heuristic:* a heuristic scheduler can be judged against an exact formulation only if both use the same conflict relation and constraints; otherwise its gains cannot be attributed to the design. *(C3) Reliability and handover accounting:* protected flows require the duplicate-delivery reliability to be constrained end to end, and the cost of switching to a shadow schedule (detection, computation, dissemination, and activation) has to be measured rather than assumed to be zero.

The contributions are fourfold:

1. An explicit resilient-scheduling formulation: We state the resource-allocation problem underlying DL-IRS as a mixed-integer linear program (MILP) over TSCH cells, subject to flow-conservation, half-duplex, conflict-avoidance, precedence/deadline, reliability, and node-disjoint backup-path constraints, and we prove that the underlying decision problem is NP-complete. The formulation constrains the end-to-end duplicate-delivery reliability of protected flows and expresses every objective term in closed linear form (Section 3.2), which addresses C2 and C3.
2. A separately specified allocation heuristic (CASA): Conflict-Aware Spatial-Spectral Allocation (CASA) is defined independently of the space–time conflict graph (STCG) that it consumes. We give its conflict construction, scoring function, pseudocode, and computational-complexity analysis.
3. A dual-loop resilience mechanism with measurable timing components: We specify the inner loop’s minimum-perturbation channel remapping and the outer loop’s shadow-schedule activation as separate algorithms with explicit trigger conditions, escalation criteria, and timing components. The separation of recovery scope, with explicit escalation criteria, addresses C1.
4. A reproducible evaluation methodology: We document the simulator configuration, topology, traffic, interference model, and controller parameters required to reproduce every reported number, supporting the evaluation with an optimality-gap analysis against an exact MILP solver.

Relation to existing mechanisms. Local channel remapping, shadow (backup) scheduling, centralized SDN control, and conflict-aware allocation are each established building blocks, and DL-IRS does not claim novelty for any of them in isolation. The contribution lies in (i) the formal separation of recovery scope with explicit escalation criteria, so that transient degradation does not trigger path-level reconfiguration and persistent failure does not wait for an exhaustive channel search; (ii) the use of one conflict relation in both the exact MILP and the CASA heuristic, which makes the heuristic auditable against an exact oracle; and (iii) an accounting of the handover cost that reports the full detection-to-activation chain instead of assuming a free switch. We note that the reported experiments evaluate the compute-on-trigger branch, while the proactive reservation phase (Phase A) remains an architectural specification rather than an experimentally validated claim. The ablation study in Section 4.6 quantifies what these elements add beyond a plain combination of the building blocks: removing either loop lowers the PDR at SNR = 0 dB by more than 22 percentage points, and removing CASA lowers it by 15.3 percentage points.

Based on Cooja/Contiki-NG simulation, DL-IRS sustains a PDR of 96.8% under an SNR = 0 dB narrowband-interference condition, an absolute improvement of 35.3 percentage points over standard 6TiSCH scheduling (61.5%) under the same topology and traffic. Section 4 reports recovery timing, spatial-reuse, control-overhead, and energy/lifetime results using explicitly defined metrics, and Section 4.7 states the scope and limitations of each claim.

Table 1 summarizes the structural distinction between the two loops along seven dimensions. Each row is independently verifiable from the algorithm specifications in Section 4: the loops differ in their trigger conditions, decision variables, spatial scope, scheduling principle, objective priority, controller action, and fallback behavior.

## 2. Literature Review

### 2.1. Foundations of Deterministic Communication in IIoT

The realization of Industry 4.0 depends heavily on the deterministic communication capabilities of the Industrial Internet of Things (IIoT). Within this landscape, the IEEE 802.15.4-TSCH protocol has emerged as a de facto standard for mission-critical applications, leveraging Time Division Multiple Access (TDMA) and channel hopping to combat multipath fading and interference. The TSCH mode is specified in IEEE 802.15.4-2020 [1], and the IETF 6TiSCH architecture builds on it with IPv6 integration and schedule-management mechanisms [2]. De Guglielmo et al. [3] provided a foundational survey on the IEEE 802.15.4e standard, detailing the operational principles of the TSCH MAC layer. Queiroz et al. [4] delivered a comprehensive survey and systematic mapping of industrial wireless sensor networks, categorizing deployment topologies and coexistence challenges that define modern IWSN environments. As noted in the surveys by Balbi et al. [5] and Urke et al. [6], while TSCH provides a robust foundation, the increasing density of industrial deployments necessitates further enhancements in scheduling efficiency and interference mitigation. The 6TiSCH stack, which integrates IPv6 over the TSCH mode, extends this capability by providing a standardized sub-layer for resource management. To route data packets within these low-power lossy architectures, Ghanbari et al. [7] provided a comprehensive survey on the Routing Protocol for Low-Power and Lossy Networks (RPL), particularly its adaptations within dynamic and mobile IoT environments. As industrial environments become more dynamic, however, the gap between standard protocol specifications and the requirements for low-disruption reliability continues to widen—a gap this work addresses by formulating the scheduling problem explicitly rather than only architecturally.

### 2.2. Limitations of Conventional Scheduling Paradigms

Conventional scheduling paradigms in IIoT have historically focused on static or reactive mechanisms. Early foundational work by Saifullah et al. [8] established the principles of real-time scheduling in WirelessHART networks, yet these approaches often struggle with bursty traffic patterns prevalent in modern smart factories. Raut et al. [9] developed a priority-based self-organized MAC protocol aimed at handling critical data traffic in real-time WSN applications. Kalita et al. [10] investigated resource-allocation schemes designed to overcome centralized bottlenecks and improve throughput and reliability metrics in 6TiSCH networks. Kalita and Gurusamy [11] proposed an on-the-fly autonomous slot-allocation mechanism for 6TiSCH-based industrial IoT networks, allowing nodes to dynamically adjust scheduling configurations without continuous central intervention. Lee and Chung [12] showed that under volatile traffic conditions, standard negotiation techniques often incur significant latency and packet loss, and Lee et al. [13] investigated cell-scheduling techniques to enhance bidirectional throughput in TSCH networks. Aimaretto and Dujovne [14] introduced the BDPC framework, which explicitly bounds application-layer delay within 6TiSCH networks for critical IIoT deployments. The same authors also proposed disjoint leaf-based MPLS-like tunnels to enhance end-to-end determinism and reliability [15], which is closely related to the node-disjoint protected routes adopted here. Zhang et al. [16] introduced a Kalman-filter scheduling mechanism for 6TiSCH networks that adapts to bursty traffic conditions.

Traditional reactive schedulers, however, trigger resource reallocation only after a link failure is detected, which is inherently unsuitable for closed-loop control systems where any interruption can propagate into operational failure. Hybrid proactive schedulers, such as the HPS framework of Tabouche et al. [17], pre-emptively select adaptive channels but do not separate transient channel-level recovery from persistent path-level recovery, and typically lack the global coordination required to bound signaling overhead in large-scale networks—a gap this work targets directly with the dual-loop separation formalized in Section 3 and empirically isolated in the ablation study of Section 4.6. Spatial reuse is likewise exploited in coordinated multi-AP WLANs, where coordinated spatial reuse has been analyzed for its throughput and latency gains [18,19]; CASA applies the same principle to TSCH cells under an explicit conflict model.

### 2.3. SDN-Based Architectures and Intelligent Scheduling

Parallel to architectural advances, artificial intelligence (AI) and machine learning (ML) have introduced a further dimension to TSCH scheduling. Jurado-Lasso et al. [20] introduced HRL-TSCH, a hierarchical reinforcement-learning framework that optimizes scheduling decisions from environmental state. Ben Yaala et al. [21] explored reinforcement-learning models to optimize TSCH scheduling matrices, demonstrating the capacity of learned agents to adapt to dynamic IIoT requirements. While ML-based approaches show promise in adapting to complex electromagnetic interference, their deployment is often hindered by computational cost and limited decision-time interpretability, which complicates verifying compliance with hard deadline and reliability contracts. As Mtowe and Kim argued [22], edge-computing-enabled communication is important for low-latency control but must be balanced against the finite energy budgets of field-deployed sensor nodes. This motivates the present work’s use of an explicit, auditable optimization formulation and a deterministic heuristic rather than a learned policy, while leaving a learning-augmented variant as future work.

Table 2 situates DL-IRS relative to representative prior mechanisms along the dimensions that most directly determine resilience behavior: whether recovery is reactive or proactive, whether local and global recovery scopes are distinguished, and whether the resource model is formally specified. Section 4.6.3 compares DL-IRS with HPS and HRL-TSCH in more detail.

## 3. System Model and Problem Definition

### 3.1. Network, Traffic, and Resource Model

Consider a directed IIoT communication graph G=(V,E), where V is the set of TSCH devices including the gateway and E⊆V×V is the set of feasible directed wireless links. Time is divided into a periodically repeated slotframe T={0, …, T−1}, and the available channel-offset set is C={0, …, C−1}. A TSCH cell is a pair (t, c)∈T×C. The physical channel used by a cell follows the TSCH hopping function h(t, c, ASN).

Let F denote the set of application flows. Each flow f∈F is described by a source $s_{f}$, destination $d_{f}$, period $P_{f}$, relative deadline $D_{f}^{max}$, minimum end-to-end reliability $\rho_{f}^{min}$, payload length $L_{f}$, and priority $w_{f}$. Critical flows use two node-disjoint route copies, indexed by $q\in Q_{f}$.

For each link e, channel offset c, and observation window r, the controller estimates a packet reception ratio $p_{e,\;c}\left(r\right)$, an expected transmission energy $\epsilon_{e,\;c}\left(r\right)$, and an interference state from measured link statistics. The estimate may use the exponentially weighted moving-average (EWMA) predictor:

(1) $${\hat{p}}_{e,\;c}\left(r\right)=\left(1-\lambda \right)\cdot {\hat{p}}_{e,\;c}\left(r-1\right)+\lambda \cdot p_{e,\;c}^{obs}\left(r\right),\;0\leq \lambda \leq 1$$

The model distinguishes protocol conflicts from radio interference. Two directed links e=(i, j) and $e^{\prime }=\left(u,\;v\right)$ are conflicting when they share an endpoint, when one transmitter can interfere with the other receiver, or when their simultaneous activation violates the adopted physical-interference condition. Let $\chi_{e,\;e^{\prime }}\in \left\{0,\;1\right\}$ denote this link-conflict relation; its full construction—combining node-sharing, protocol carrier-sense conflict, and physical SINR conflict—reused unchanged in the MILP constraints below, so that the conflict definition used by the exact formulation and the definition used by the CASA heuristic are provably identical.

The controller maintains an active schedule S and, for critical traffic, a standby shadow schedule $S^{\prime }$. The inner loop modifies a restricted subset of channel-offset assignments while preserving time offsets and routes. This separation is what makes the low-disruption behavior claimed in Section 1 explicit and measurable rather than a description of intent.

### 3.2. Resilient Scheduling MILP

#### 3.2.1. Modeling Note on Reliability Linearization

A weighted objective that treats reliability as a product of per-link PDRs is nonlinear. For a true MILP, we instead use a precomputed additive reliability penalty:

(2) $$l_{e,\;c}=-\log\left(\max\left\{p_{e,\;c},\;\varepsilon \right\}\right),$$

where ε>0 prevents a numerical singularity at $p_{e,\;c}=0$. A route-reliability requirement $\prod p_{e,\;c}\geq \rho_{f}^{min}$ then becomes the linear constraint $\sum l_{e,\;c}x\leq -\log\rho_{f}^{min}$ (constraint (10) below). This linearization assumes link-level reliability outcomes are conditionally independent given the schedule; we state this explicitly as a modeling approximation and evaluate its robustness under temporally correlated interference in the interference-model-sensitivity experiment.

#### 3.2.2. Sets, Parameters, and Decision Variables

Table 3 summarizes the primary notation and parameters used in the mixed-integer linear programming (MILP) formulation for resilient 6TiSCH scheduling.

#### 3.2.3. Objective

The exact form of the delay term depends on whether each packet is constrained within one slotframe or across a hyperperiod. In this work, we enforce a single-slotframe periodic schedule and report this as an explicit modeling assumption; extending to multi-slotframe hyperperiod scheduling is future work.

(3) $$\min\Phi =\sum_{f\in F}w_{f}\left[\alpha \cdot \left(\frac{d_{f}}{D_{f}^{max}}\right)+\beta \cdot u_{f}+\gamma \cdot \left(\frac{e_{f}}{E_{f}^{bud}}\right)+\eta \cdot o_{f}\right],$$

where $d_{f}$ is the scheduled end-to-end delay surrogate, $u_{f}$ is a nonnegative reliability-violation slack, $e_{f}$ is energy consumption, and $o_{f}$ denotes control/update cost. The weights α,β, γ, η≥0 are normalized per term. Because reliability and deadlines are mandatory for critical traffic, we enforce them as hard constraints (constraints (10) and (11) below) for critical flows rather than allowing the optimizer to trade them away through the slack variable $u_{f}$; $u_{f}$ applies only to best-effort flows. In all reported experiments, (α, β, γ, η) = (0.4, 0.3, 0.2, 0.1), which sums to one: the delay term receives the largest weight because deadline preservation is the primary requirement of critical flows, the reliability-slack term the second largest, and the energy and update-cost terms act as secondary regularizers. The sensitivity of the schedule to these values is analyzed in Section 4.6.2.

(4) $$y_{e,\;t,\;c}\geq x_{f,\;q,\;e,\;t,\;c},\;\forall f,\;q,\;e,\;t,\;c;$$

(5) $$e_{f}=\sum_{q\in Q_{f}}\sum_{e\in E}\sum_{t\in T}\sum_{c\in C}\varepsilon_{e,\;c}\cdot x_{f,\;q,\;e,\;t,\;c}$$

The control/update cost counts the cell changes with respect to the currently installed schedule, whose indicator values $x_{f,q,e,t,c}^{0}$ are constants:

(6) $$o_{f}=\sum_{q\in Q_{f}}\sum_{e\in E}\sum_{t\in T}\sum_{c\in C}\left[x_{f,q,e,t,c}\left(1-x_{f,q,e,t,c}^{0}\right)+x_{f,q,e,t,c}^{0}\left(1-x_{f,q,e,t,c}\right)\right]$$

Because the indicators are binary, (6) is linear in the decision variables. The delay surrogate $d_{f}$ and the reliability slack $u_{f}$ are defined in (13) and (16), so every term of (3) is available in closed linear form.

#### 3.2.4. Core Constraints

- Path-flow conservation:

For every flow copy (f, q) and every node v,

(7) $$\sum_{e\in \delta^{+}\left(v\right)}z_{f,\;q,\;e}-\sum_{e\in \delta^{-}\left(v\right)}z_{f,\;q,\;e}=\{\begin{array}{c} 1,\;v=s_{f};\\ -1,\;v=d_{f};\\ 0,\;\mathrm{otherwise}. \end{array}$$

A link is allocated exactly one cell in a flow copy if and only if it is selected for that copy, i.e., the number of cells allocated to link e in copy q equals its selection indicator:

(8) $$\sum_{t\in T}\sum_{c\in C}x_{f,q,e,t,c}=z_{f,q,e},\;\forall f,q,e$$

- Half-duplex constraint:

No node may transmit and/or receive on more than one incident link in the same slot. For every v∈V and t∈T,

(9) $$\sum_{c\in C}\sum_{e\in \delta^{+}\left(v\right)\cup \delta^{-}\left(v\right)}y_{e,\;t,\;c}\leq 1$$

This work assumes a single half-duplex radio per node; if the target hardware provides multiple radios, the right-hand side is replaced by the verified radio count.

- Interference and conflict avoidance:

For every pair $e,\;e^{\prime }$ with $\chi_{e,\;e^{\prime }}=1$, and every (t, c),

(10) $$y_{e,\;t,\;c}+y_{e^{\prime },\;t,\;c}\leq 1$$

This constraint is the primary bridge between CASA and the MILP: both use the identical conflict relation $\chi_{e,\;e^{\prime }}$, so a schedule that CASA certifies conflict-free also satisfies (9), and vice versa.

- Precedence and delay:

For consecutive hops h and h−1 on a selected path, the assigned slot offsets must respect topological precedence within the slotframe, and the accumulated slot-distance contributions $\delta_{t,\;t^{\prime }}$ over the path bound the delay surrogate $d_{f}$ used in the objective. We enforce this with the following big-M precedence linearization, with M=T. The slot offset of link e in copy q of flow f is

(11) $$t_{f,q,e}=\sum_{t\in T}\sum_{c\in C}t\;x_{f,q,e,t,c}$$

For consecutive links e=(u,v) and e′=(v,w) on a copy, the precedence constraint is

(12) $$t_{f,q,e\prime }\geq t_{f,q,e}+1-M\left(2-z_{f,q,e}-z_{f,q,e^{\prime }}\right),\;\forall f,q,\;\forall \left(e,e^{\prime }\right)\;\mathrm{consecutive}$$

If both links are selected, (12) forces $t_{f,q,e\prime }\geq t_{f,q,e}+1$; otherwise the constraint is inactive because every slot offset lies in [0,T−1]. With slot duration $T_{s}$ and packets released at the start of the slotframe, the delay surrogate conservatively bounds the completion slot of the last hop of every copy:

(13) $$d_{f}\geq T_{s}\left(t_{f,q,e}+1\right)-T_{s}M\left(1-z_{f,q,e}\right),\;\forall f,q,\;\forall e=\left(v,d_{f}\right)$$

For critical flows, $d_{f}\leq D_{f}^{max}$ is imposed as a hard constraint.

- Reliability constraints:

Using the linearized penalty of (2), a per-copy route-reliability requirement with per-copy target $\rho_{f}^{cp}$ (defined below) is

(14) $$\sum_{e,c}l_{e,c}\;x_{f,q,e,t,c}\leq -log\rho_{f}^{cp},\;\forall f,q$$

For protected flows with duplicate delivery over two node-disjoint copies, the requirement is stated end to end. Let $\rho_{f,q}=exp\left(-\sum l_{e,c}x_{f,q,e,t,c}\right)$ denote the reliability of copy q. Under the conditional-independence assumption of Section 3.2.1, the duplicate-delivery reliability of flow f is $1-\left(1-\rho_{f,1}\right)\left(1-\rho_{f,2}\right)$, and the end-to-end requirement is

(15) $$1-\left(1-\rho_{f,1}\right)\left(1-\rho_{f,2}\right)\geq \rho_{f}^{min}$$

Because (15) is bilinear in the copy reliabilities, it is enforced through a linear sufficient condition: each copy must satisfy the symmetric per-copy target $\rho_{f}^{cp}=1-\sqrt{1-\rho_{f}^{min}}$ in (10), which guarantees ${\left(1-\rho_{f}^{cp}\right)}^{2}=1-\rho_{f}^{min}$. For single-copy flows, $\rho_{f}^{cp}=\rho_{f}^{min}$. For example, $\rho_{f}^{min}=0.99$ yields $\rho_{f}^{cp}=0.90$ per copy. The condition is conservative when the two copies differ in quality; asymmetric allocations can be expressed with a piecewise-linear approximation of (15) at the cost of additional binary variables. For best-effort flows, (10) is relaxed with the nonnegative slack $u_{f}$:

(16) $$\sum_{e,c}l_{e,c}\;x_{f,q,e,t,c}\leq -log\rho_{f}^{min}+u_{f},\;\forall f\;\text{best-effort},\;\forall q$$

The measured end-to-end duplicate-delivery reliability is additionally reported empirically in Section 4. Because node-disjoint routes can still be correlated through shared interference sources, the independence assumption is stated as a modeling approximation and examined in the interference-model sensitivity experiment.

- Node-disjoint backup paths:

For critical flow f, copies q=1 and $q^{\prime }=2$ must not share intermediate nodes:

(17) $$\sum_{e\in \delta^{+}\left(v\right)}z_{f,\;1,\;e}+\sum_{e\in \delta^{+}\left(v\right)}z_{f,\;2,\;e}\leq 1,\;\forall v\in V\backslash \left\{s_{f},\;d_{f}\right\}$$

In this work, the sink receives the two copies in separate slots, so the half-duplex condition (9) is retained at the destination.

- Shadow-schedule reservation:

For critical flows, standby cells are reserved only when they do not conflict with active cells that cannot be preempted:

(18) $$b_{f,\;q,\;e,\;t,\;c}+y_{e^{\prime },\;t,\;c}\leq 1,\;\forall \chi_{e,\;e^{\prime }}=1$$

The formulation supports two reservation semantics; this work adopts the dedicated reservation for all reported results:

- Dedicated reservation: backup cells remain idle during normal operation; highest predictability, lower utilization.
- Preemptible reservation: lower-priority traffic may use backup cells but must be evicted before the activation ASN; higher utilization at the cost of a nonzero preparation/control requirement.

### 3.3. Computational Complexity and Exact-Solver Role

**Proposition** **1.** *The decision version of the resilient TSCH scheduling problem is NP-complete, even in the restricted case of a single slotframe, one channel offset, one-hop flows, no energy objective, and no backup-path requirement.*

**Proof.**  Membership in NP follows because a candidate schedule can be verified in polynomial time by checking each flow assignment against the half-duplex condition (9) and the conflict constraint (10). For NP-hardness, we reduce from graph coloring. Given an arbitrary graph H=(U, A) and an integer K, create one one-hop TSCH transmission for each vertex in U. For every edge (u, v)∈A, configure the corresponding transmissions as mutually conflicting in the conflict graph (χ=1). Use one channel offset and K available time slots. A feasible TSCH schedule assigns each transmission one slot such that conflicting transmissions do not share a slot; this is exactly a proper K-coloring of H. A feasible schedule therefore exists if and only if H is K-colorable. Since graph coloring is NP-complete, the restricted scheduling problem—and hence the general resilient TSCH scheduling problem, which contains it as a special case—is NP-hard. □

Because the problem is NP-hard, we do not claim a polynomial approximation ratio for the CASA heuristic (Section 3.4) unless one is proven under explicit assumptions; no such proof is offered here. Instead, we evaluate the heuristic empirically against an exact MILP oracle on small instances (Section 4.5, experiment E5), reporting the objective gap and runtime rather than an asymptotic guarantee. The MILP itself is used only as a small-scale correctness and quality oracle: at the network sizes evaluated in Section 4 (50–150 nodes), exact solution is not attempted, and CASA is the schedule-construction mechanism used in all reported experiments outside Section 4.5.

### 3.4. DL-IRS Architecture, CASA, and Dual-Loop Execution

**Figure 1:**
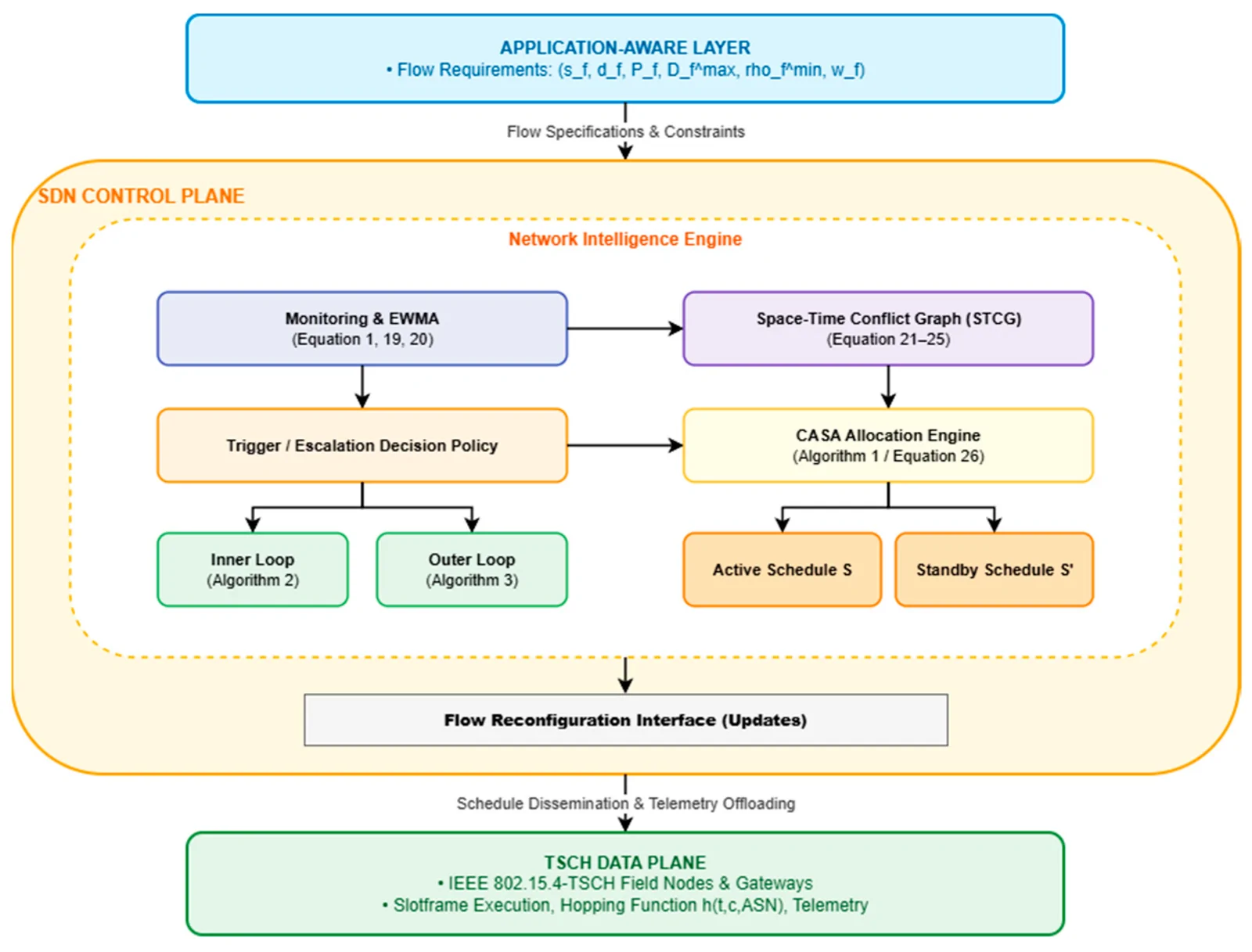
System architecture and end-to-end controller workflow of DL-IRS.

The DL-IRS architecture adopts a hierarchical design that decouples industrial application requirements from the underlying deterministic transmission mechanisms. The architecture is vertically structured into three functional planes: an Application-Aware Layer that identifies per-flow QoS requirements, an SDN Control Plane that centralizes scheduling authority in a Network Intelligence Engine, and a TSCH Data Plane of field-deployed IEEE 802.15.4-TSCH nodes. The Network Intelligence Engine maintains a space–time conflict graph (STCG) recording cell occupancy and conflict relations across the network, computes schedules using the CASA allocation procedure, and disseminates incremental updates through a Flow Reconfiguration Interface using compact update commands rather than full schedule replacement (As shown in Figure 1).

#### 3.4.1. Monitoring, Prediction, and Trigger Policy

Each TSCH node maintains a localized link-quality table and reports the packet reception ratio and received-signal metrics to the controller once per slotframe using dedicated advertising slots. The controller maintains the EWMA-smoothed reliability estimate ${\hat{p}}_{e,\;c}\left(r\right)$ of Equation (1) for every monitored link-channel pair.

For each active link-channel pair (e, c), define the predicted reliability margin

(19) $$B_{e,\;c}\left(r\right)={\hat{p}}_{e,\;c}\left(r\right)-\rho_{e}^{min}$$

Define a trend estimate over a window of W monitoring epochs,

(20) $$\Delta {\hat{p}}_{e,\;c}\left(r\right)=\frac{{\hat{p}}_{e,\;c}\left(r\right)-{\hat{p}}_{e,\;c}\left(r-W\right)}{W}$$

The controller enters the warning state when $B_{e,\;c}\left(r\right)<\Gamma_{w}$ or $\Delta {\hat{p}}_{e,\;c}\left(r\right)<-\kappa$. The controller escalates to the outer loop when at least one of the following holds: (i) no feasible local remapping exists; (ii) the warning condition persists for K epochs after remapping; (iii) the node or link is unavailable; or (iv) the end-to-end reliability/deadline prediction of a critical flow violates its contract.

The margin threshold $\Gamma_{w}$ is reported as an absolute PRR margin (e.g., $\Gamma_{w}=0.05$) rather than as an unqualified percentage of $\rho_{min}$, to avoid unit ambiguity. Section 4.6.2 (experiment E7) reports a sensitivity sweep over λ, $\Gamma_{w}$ and K rather than asserting a single fixed operating point as universally optimal.

Scope of the predictive claim. The term “predictive” is used in a restricted, operational sense. The warning test (19) fires while the smoothed PRR is still $\Gamma_{w}$ above the contract bound, and the trend test (20) fires when the PRR falls faster than κ per epoch, so a warning can precede an actual contract violation; neither test forecasts arbitrary interference dynamics. With λ=0.3 the EWMA has an effective memory of (2−λ)/λ≈5.7 epochs, and one epoch equals one slotframe (101 slots × 10 ms ≈ 1.01 s). A step degradation of size s is flagged in the first epoch whenever λs exceeds the remaining margin $B_{0}-\Gamma_{w}$, where $B_{0}$ is the margin before the step, whereas small or gradual degradations are detected after a lag of the order of the effective memory. Interference that appears and disappears faster than about one epoch is not predicted; it is absorbed by channel hopping and MAC retransmissions and, if it persists, handled reactively by the inner loop within K epochs. The trigger is therefore a trend-and-margin early-warning mechanism whose adequacy for rapidly varying interference depends on λ, $\Gamma_{w}$, and K; this dependence is evaluated in Section 4.6.2.

#### 3.4.2. CASA: Conflict-Aware Spatial-Spectral Allocation

We use a single consistent expansion throughout: CASA is Conflict-Aware Spatial-Spectral Allocation. CASA is not a label attached to the STCG construction procedure; the STCG/conflict graph is the resource model, and CASA is the allocation algorithm that consumes that model to produce a schedule.

For directed links e=(i, j) and $e^{\prime }=\left(u,\;v\right)$, define

(21) $$\chi_{e,\;e^{\prime }}=\max\left\{\chi_{e,\;e^{\prime }}^{node},\;\chi_{e,\;e^{\prime }}^{pri},\;\chi_{e,\;e^{\prime }}^{phy}\right\}$$

The three components are

(22) $$\chi_{e,\;e^{\prime }}^{node}=1\left[\left\{i,\;j\right\}\cap \left\{u,\;v\right\}\neq 0\right]$$

(23) $$\chi_{e,\;e^{\prime }}^{pri}=1\left[P_{i\to v}^{rx}\geq \Gamma_{v}VP_{u\to j}^{rx}\geq \Gamma_{j}\right]$$

(24) $$\chi_{e,\;e^{\prime }}^{phy}=1\left[\frac{P_{i\to j}^{rx}}{N_{0}+P_{u\to j}^{rx}}<{SINR}_{min}\;V\frac{P_{u\to v}^{rx}}{N_{0}+P_{i\to v}^{rx}}<{SINR}_{min}\right]$$

where 1[·] is an indicator function, $P_{a\to b}^{rx}$ is predicted received power, $\Gamma_{b}$ is the carrier-sense/interference threshold at node b, $N_{0}$ is noise power, and ${SINR}_{min}$ is the decoding threshold. This is exactly the $\chi_{e,\;e^{\prime }}$ used in MILP constraints (10) and (18); the two are defined once and referenced identically in both places. Where Cooja cannot supply calibrated received-power values for this SINR-based model, we substitute an explicitly stated protocol-interference approximation based on communication and interference radii, and we label it as such rather than describing it as a physical SINR model.

The reuse distance between links is

(25) $$d_{reuse}\left(e,\;e^{\prime }\right)=\min\left\{d\left(i,\;v\right),\;d\left(u,\;j\right),\;d\left(i,\;u\right),\;d\left(j,\;v\right)\right\}$$

A candidate reuse pair is admissible only if $\chi_{e,\;e^{\prime }}=0$ and $d_{reuse}\left(e,\;e^{\prime }\right)\geq d_{min}^{reuse}$.

For each candidate cell (t, c), CASA scores a feasible allocation of link e by

(26) $$Score\left(e,\;t,\;c\right)=\omega_{1}{\hat{p}}_{e,\;c}-\omega_{2}\left(\frac{I_{e,\;t,\;c}}{I_{max}}\right)-\omega_{3}\left(\frac{Q_{e,\;t}}{Q_{max}}\right)+\omega_{4}\left(\frac{\Delta SR\left(e,\;t,\;c\right)}{{SR}_{max}}\right)-\omega_{5}\left(\frac{U\left(e,\;t,\;c\right)}{U_{max}}\right)$$

where $I_{e,\;t,\;c}$ is predicted interference, $Q_{e,\;t}$ is queue urgency/deadline slack, ΔSR is the marginal spatial-reuse gain, and U is control/update cost. Critical traffic receives lexicographic priority over best-effort traffic in the allocation order (Algorithm 1, line 2); the weighted score in (26) governs ordering among candidate cells for a given hop only after that lexicographic priority is applied. The complete allocation procedure is formalized in Algorithm 1.

Weight selection. The CASA weights are set to $\left(\omega_{1},\omega_{2},\omega_{3},\omega_{4},\omega_{5}\right)=$ (0.4, 0.2, 0.2, 0.1, 0.1), which sums to one. The ordering reflects the priority structure of the design: the predicted reliability of the candidate cell ($\omega_{1}$) dominates because it governs the reliability contract; interference avoidance ($\omega_{2}$) and deadline urgency ($\omega_{3}$) are second-order and equally weighted; spatial-reuse gain ($\omega_{4}$) and update cost ($\omega_{5}$) act as tie-breakers among cells that already satisfy feasibility and reliability. Section 4.6.2 reports a one-factor-at-a-time sensitivity analysis in which each weight is perturbed by ±50% and the remaining weights are renormalized.

We report two distinct spatial-reuse metrics: Average concurrent-link-per-slot (ACLS) is

(27) $$ACLS=\frac{1}{T}\sum_{t\in T}\left|\left\{e:y_{e,\;t,\;c}=1\;for\;some\;c\right\}\right|$$

Spectral spatial-reuse efficiency (SSRE) is the normalized cell-occupancy ratio

(28) $$SSRE=\frac{\sum_{t\in T}\sum_{c\in C}\sum_{e\in E}y_{e,\;t,\;c}}{T\times C}$$

In (28), only active cells are counted; shadow reservations (variables b) are idle until activation, are excluded from the numerator, and are reported separately. Because each selected link is bound to exactly one cell (8) and at most one incident link of a node can be active in a slot (9), a link cannot occupy two channel offsets in the same slot; the numerator of (28) therefore equals T×ACLS and SSRE=ACLS/C.

Where DL-IRS is compared against a baseline in relative terms, we report the relative SSRE gain explicitly as

(29) $${SSRE}_{gain}\left(\%\right)=100\times \frac{{SSRE}_{DL-IRS}-{SSRE}_{baseline}}{{SSRE}_{baseline}}$$

and Section 4.3 states, for every reported percentage, whether it is this relative gain, an absolute occupancy value from (28), or an absolute percentage-point difference. In this paper, “SSRE gain” always denotes the relative gain (29), “SSRE” the absolute ratio (28), and percentage-point differences are always labeled as such.
**Algorithm 1:** CASA—Conflict-Aware Spatial-Spectral AllocationInput: Graph G = (V,E), flow copies R, active schedule S, link estimates p_hat, interference estimates I, slot set T, channel-offset set C Output: Conflict-free schedule S and candidate remapping set A 1: Build link-conflict graph Gc = (E, Ec) using chi_node, chi_pri, chi_phy [Equations (15)–(18)] 2: Sort flow copies by lexicographic key: (criticality descending, deadline slack ascending, reliability deficit descending) 3: for each flow copy r in sorted order do 4:      for each required hop e in r do 5:          F <- empty set 6:          for each candidate cell (t,c) satisfying hop precedence do 7:              if halfDuplex(e,t,S) and noConflict(e,t,c,S,Gc) and reliabilityFeasible(e,c) then 8:                  Compute Score(e,t,c) [Equation (20)] 9:                  Add (t,c,Score) to F 10:              end if 11:          end for 12:          if F is empty then 13:              invoke bounded repair: relocate lowest-priority movable allocation 14:              recompute F; if still empty, return INFEASIBLE 15:          end if 16:          Select argmax_(t,c,Score) in F; assign e to (t,c); update S 17:      end for 18: end for 19: A <- channel-remapping candidates for active links with low p_hat and feasible local alternatives 20: return S, A

#### 3.4.3. Inner-Loop Channel Remapping

The inner loop is predicated on the principle of minimum perturbation: it modifies only the channel offset of an affected link, holding the slot offset, route, and all other schedule entries fixed. On entering the warning state, the controller attempts minimum-perturbation local recovery as specified in Algorithm 2, changing only the channel offset for the affected link while holding the slot offset and routing fixed. If no feasible local channel offset exists or if the link quality remains below the threshold for K consecutive epochs after remapping, the controller escalates to the outer loop (Algorithm 2, lines 3 & 8).
**Algorithm 2:** Inner-Loop Minimum-Perturbation Channel RemappingInput: Active schedule S, affected link e, current channel c0, candidate set A(e), activation lead time delta_ASN Output: Updated schedule S or ESCALATE 1: Estimate p_hat(e,c) and interference I(e,c) for all c in A(e) 2: F <- { c in A(e) : c is conflict-free at e’s fixed slot and p_hat(e,c) >= rho_e_min } 3: if F is empty then return ESCALATE 4: c* <- argmin_{c in F} [ interference(e,c) + updateCost(c0,c) − xi * p_hat(e,c) ] 5: Send incremental update (link ID, retained slot offset, new channel offset c*, activation ASN) 6: Verify acknowledgement or deterministic dissemination completion before activation ASN 7: Atomically switch e to c* at the activation ASN 8: Monitor for K epochs; if contract remains violated then return ESCALATE 9: return S with c0 replaced by c*

Because the slot offset is preserved (line 7), the wake-up timing of the affected nodes within the slotframe is unchanged; only the frequency used at that slot changes, via the TSCH hopping function h(t, c, ASN).

#### 3.4.4. Outer-Loop Shadow Scheduling and Atomic Activation

The outer loop addresses structural threats that the inner loop cannot resolve. Rather than reacting after the primary path fails, the controller reserves standby resources in advance and, on an early-warning trigger, instantiates and installs a standby shadow schedule $S^{\prime }$ for protected flows. When local recovery fails or persistent path degradation is detected, the controller triggers the outer-loop shadow-schedule activation procedure described in Algorithm 3. By executing the make-before-break activation, Algorithm 3 pre-validates and installs the shadow schedule $S^{\prime }$ before the target Absolute Slot Number (${ASN}_{act}$), ensuring a minimally disruptive data-plane handover (Algorithm 3, steps 5–7).

**Figure 2:**
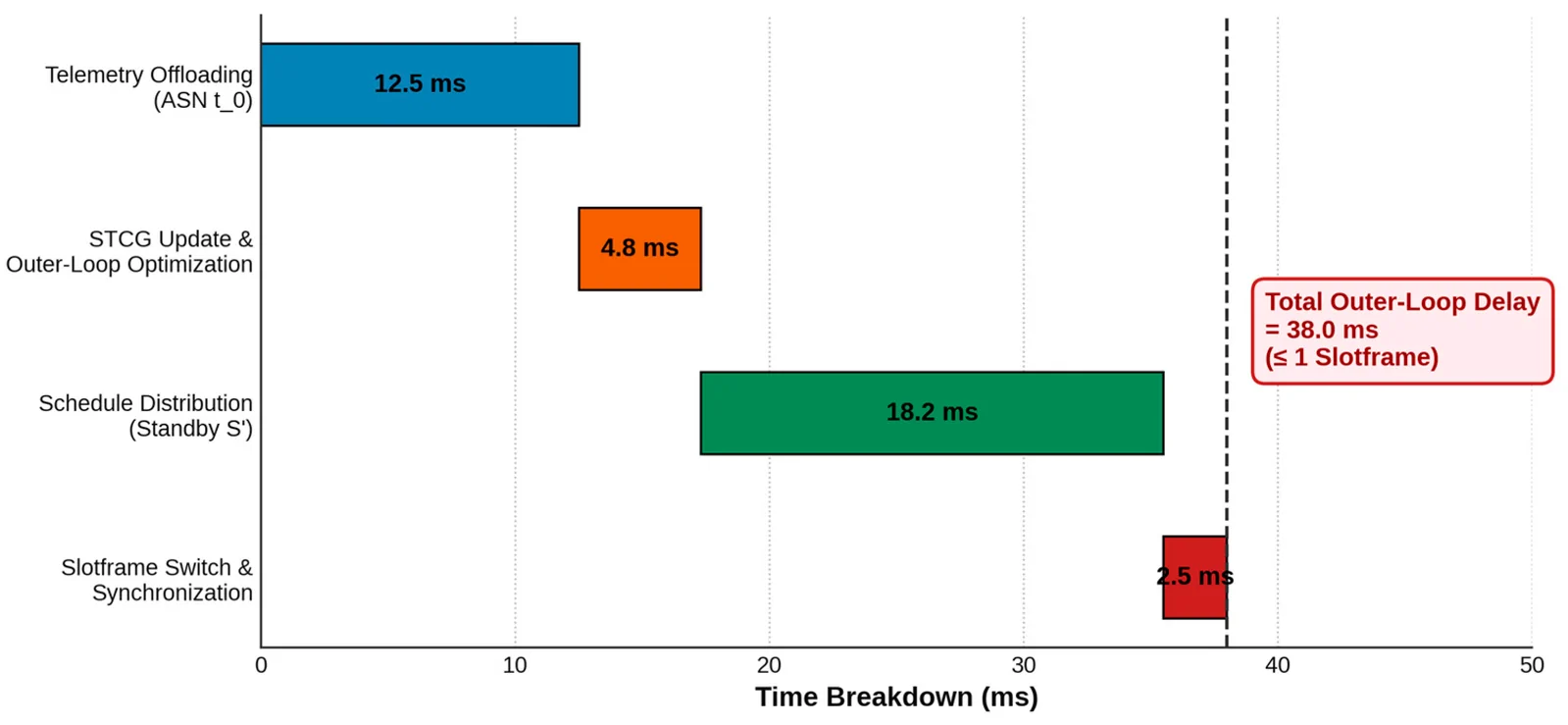
Detailed breakdown of recovery timing components during a single-slotframe outer-loop activation.

We define “minimally disruptive data-plane handover” as the operational claim supported by this mechanism: the data-plane switch at step 7 incurs no route recomputation at the activation ASN, because S′ is computed, validated, and installed before ${ASN}_{act}$ (steps 1–6). This is not a claim of zero detection-to-activation latency. Algorithm 3 executes steps 1–6 after the trigger, and Figure 2 shows that the complete chain takes 38.0 ms: telemetry offloading (12.5 ms), STCG update and outer-loop optimization (4.8 ms), schedule distribution (18.2 ms), and slotframe switch and synchronization (2.5 ms). By design, Phase A is intended to remove the path search from this chain whenever the reserved standby path is still valid. However, all reported experiments in this paper exercise exclusively the compute-on-trigger branch of step 2; the Phase A reservation-hit path is specified as part of the system architecture but is not evaluated experimentally in this paper. Consequently, the claim that make-before-break removes the path search from the recovery chain remains an architectural expectation rather than an experimentally measured result. The 38.0 ms figure is the measured chain of the compute-on-trigger branch and must not be read as the latency of the precomputed-standby case; quantifying that case requires a controlled comparison with Phase A enabled and disabled, which is left for future work (Section 5). The minimum lead time must cover the computation and dissemination stages, $\delta_{min}\geq \lceil \left(4.8+18.2\right)/10\rceil =3$ slots for the 10 ms slot duration; if installation is not confirmed in time, activation is postponed by one slotframe and, failing that, the controller falls back to break-then-make reallocation (step 6).
**Algorithm 3:** Outer-Loop Shadow-Schedule ActivationInput: Active schedule S, protected critical flows Fc, current topology and link estimates, reservation state R, minimum lead time delta_min Output: Activated shadow schedule S′ or fallback to break-then-make reallocation Phase A (background, before any trigger): A1: For each f in Fc, maintain node-disjoint candidate backup routes and reserve standby cells in dedicated mode [Equation (18)] A2: Refresh the reservations whenever topology or link estimates change by more than the warning threshold Phase B (on trigger): 1: For each f in Fc, identify the invalid primary/backup segment and residual deadline 2: If the reserved standby path of f is still valid, adopt it; otherwise run CASA (Algorithm 1) on the invalid segment to compute node-disjoint protected routes and standby cells (compute-on-trigger) 3: Construct S′ and UpdateSet = diff(S, S′) 4: Validate: half-duplex, conflict-free allocation, route disjointness, deadline, and reliability constraints [Equations (8)–(12)] 5: Select ASN_act >= ASN_now + delta_min, with delta_min >= ceil((T_opt + T_dis)/T_slot); disseminate UpdateSet and ASN_act to all affected nodes 6: Confirm installation before ASN_act; if any required installation fails, postpone ASN_act by one slotframe and retry once, otherwise invoke the break-then-make fallback (full CASA reallocation of the affected flows) 7: At ASN_act, atomically activate S′ 8: Measure packet-level recovery, schedule-installation latency, and any packet loss during the transition 9: return S′

#### 3.4.5. Controller Stage Complexity

Table 4 reports the worst-case and typical-case asymptotic complexity of each stage, with m=|E|, n=|V|, and H the maximum path hop count.

## 4. Evaluation, Discussion, and Limitations

### 4.1. Reproducible Simulation Methodology

We evaluate DL-IRS and CASA in the Cooja simulator within the Contiki-NG operating system, modeling an industrial factory floor spanning 100 m × 100 m with a node density of 50, 75, or 100 sensor nodes. We introduce narrowband-interference (NBI) sources representing overlapping IEEE 802.11b/g signals and a periodic-pulse interferer to approximate the high-EMI environment of industrial machinery. Table 5 records the full simulation configuration used in all experiments.

### 4.2. Reliability, Deadline, and Recovery Performance

**Figure 3:**
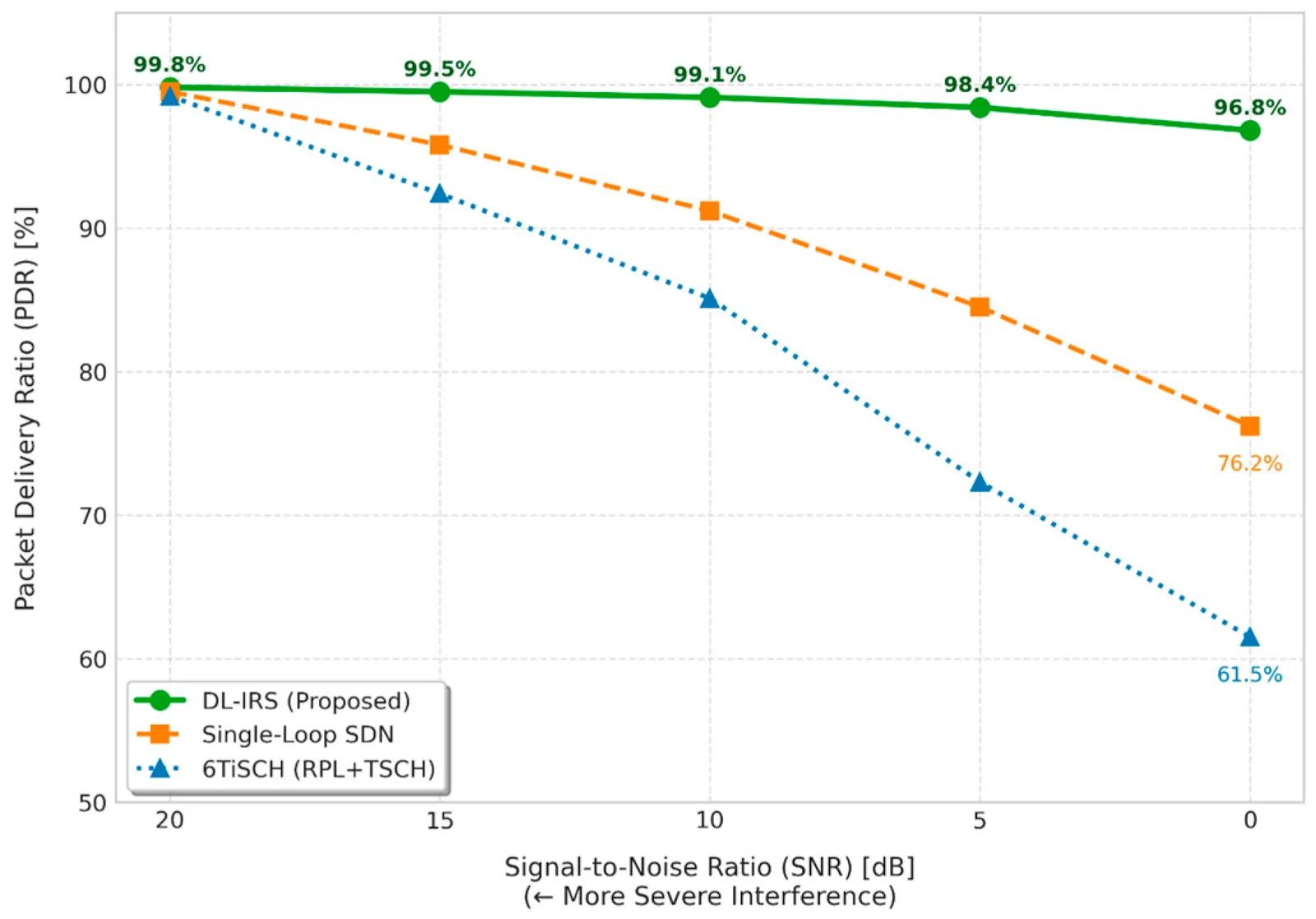
Packet delivery ratio (PDR) versus SNR, DL-IRS versus single-loop SDN and standard 6TiSCH.

Under an SNR = 0 dB narrowband-interference condition, DL-IRS sustains a packet delivery ratio of 96.8%, compared with 61.5% for standard 6TiSCH scheduling and 76.2% for a single-loop SDN baseline under the same topology and traffic (Figure 3)—an absolute improvement of 35.3 percentage points over standard 6TiSCH. The 61.5% point corresponds to standard 6TiSCH operating with the configuration listed in Table 6 (RPL with the MRHOF objective function, no interference-aware channel remapping, and at most three MAC retries) under the interference sources of Table 5 (WiFi-overlap channels 1, 6, and 11 plus periodic EMI pulses), 20 independent seeds, and the 100-node topology.

**Table 6:** Baseline implementation and configuration.

Baseline	Matching Conditions	Configuration Used
Standard 6TiSCH	Same TSCH timing, topology, traffic, radio/interference model	RPL with the MRHOF (ETX) objective function; autonomous scheduling function without interference-aware channel remapping; at most 3 MAC retries; local RPL parent switching on link degradation; no shadow resources and no controller
Reactive SDN	Same controller and monitoring substrate; recovery after confirmed failure	Failure declared after K = 3 consecutive epochs below the reliability bound; full CASA recomputation after the confirmed failure; complete update dissemination
Single-loop SDN	Same resource model but no separation between local and global actions	Warning trigger of Section 3.4.1; every trigger invokes global CASA recomputation and a path-level update (no local channel-remapping action)
HPS [17]	Same channel set, traffic, and topology	Not re-implemented in this study; qualitative comparison in Table 7
HRL-TSCH [20]	Same observation/reward/action space as reported by the original work	Not re-implemented in this study; qualitative comparison in Table 7
MILP optimum	Small networks only	Gurobi 10.0; 3600 s time limit; optimality proven for N ≤ 20; MIP gap 1.1% at N = 25; time limit reached at N = 30 (Table 8)

**Table 7:** Qualitative comparison of DL-IRS with HPS [17] and HRL-TSCH [20].

Aspect	HPS [17]	HRL-TSCH [20]	DL-IRS (This Work)
Recovery trigger	Proactive channel selection	Learned policy (reward-driven)	Trend and margin early warning with escalation
Recovery scopes	Single adaptation scope (channel)	Single learned action space	Two scopes: local channel remapping and end-to-end shadow path
Resource model	Heuristic	Implicit (reward-defined)	Explicit MILP with a conflict relation shared with CASA
Offline cost	Heuristic parameters	Training episodes and convergence	None; weights fixed once (Table 5)
Contract checking	Heuristic, no formal check	Limited decision-time interpretability	Constraint validation before activation (Equations (8)–(12))
Evaluated in this work	Not re-implemented	Not re-implemented	Yes (Section 4.2, Section 4.3, Section 4.4, Section 4.5 and Section 4.6)

**Table 8:** Optimality-gap analysis comparing CASA heuristic against exact MILP oracle.

Node Count (N)	Flow Count (F)	MILP Objective (ΦMILP)	CASA Objective (ΦCASA)	Optimality Gap (%)	MILP Runtime (s)	CASA Runtime (ms)
10	4	12.45	12.60	1.20%	4.2	1.1
15	6	18.80	19.25	2.39%	48.6	2.4
20	8	24.10	25.02	3.82%	612.0	4.2
25	10	31.50	33.10	5.08%	2840.5 (MIP Gap 1.1%)	7.8
30	12	38.20 (Time Limit)	40.45	5.89% *	>3600.0	12.5

* Note: For N=30, the MILP solver reached the maximum specified execution time limit (3600.0 s) before achieving complete convergence. The reported optimality gap (5.89%) for this instance represents an upper bound computed against the best feasible lower bound found by the solver at the time limit.

To isolate the constituent latencies of the recovery interval, Figure 2 decomposes the 38.0 ms detection-to-activation chain of the outer loop, which is well below one slotframe (101 slots × 10 ms = 1.01 s), into its operational components based on packet-level traces: telemetry offloading (12.5 ms), STCG update and outer-loop optimization (4.8 ms), schedule distribution (18.2 ms), and slotframe switch and synchronization (2.5 ms). The handover is therefore not instantaneous; it is minimally disruptive in the sense defined in Section 3.4.4. These components were measured with the compute-on-trigger branch of Algorithm 3; the Phase A reservation-hit path was not exercised.

**Figure 4:**
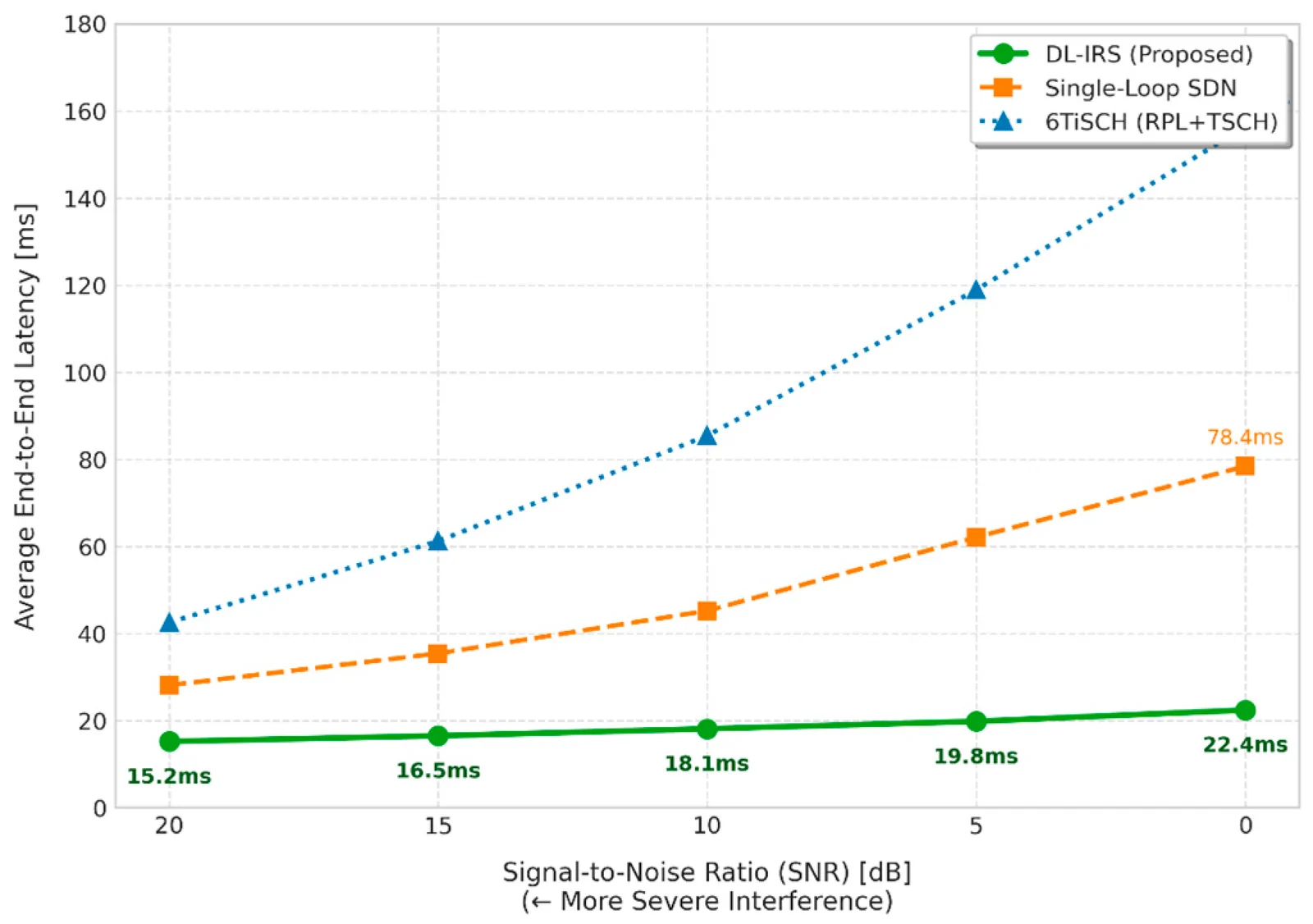
End-to-end latency versus SNR, DL-IRS versus single-loop SDN and standard 6TiSCH.

End-to-end latency is reduced relative to both baselines across the tested SNR range (Figure 4): at SNR = 0 dB, DL-IRS achieves a mean end-to-end latency of 22.4 ms versus 78.4 ms for the single-loop SDN baseline, a reduction of 71.4% at that operating point. Mission-critical packets are delivered within the configured deadline $D_{f}^{max}$ in 99.2% of simulated critical-flow packets under the evaluated conditions.

#### Recovery-Timing Decomposition

**Figure 5:**
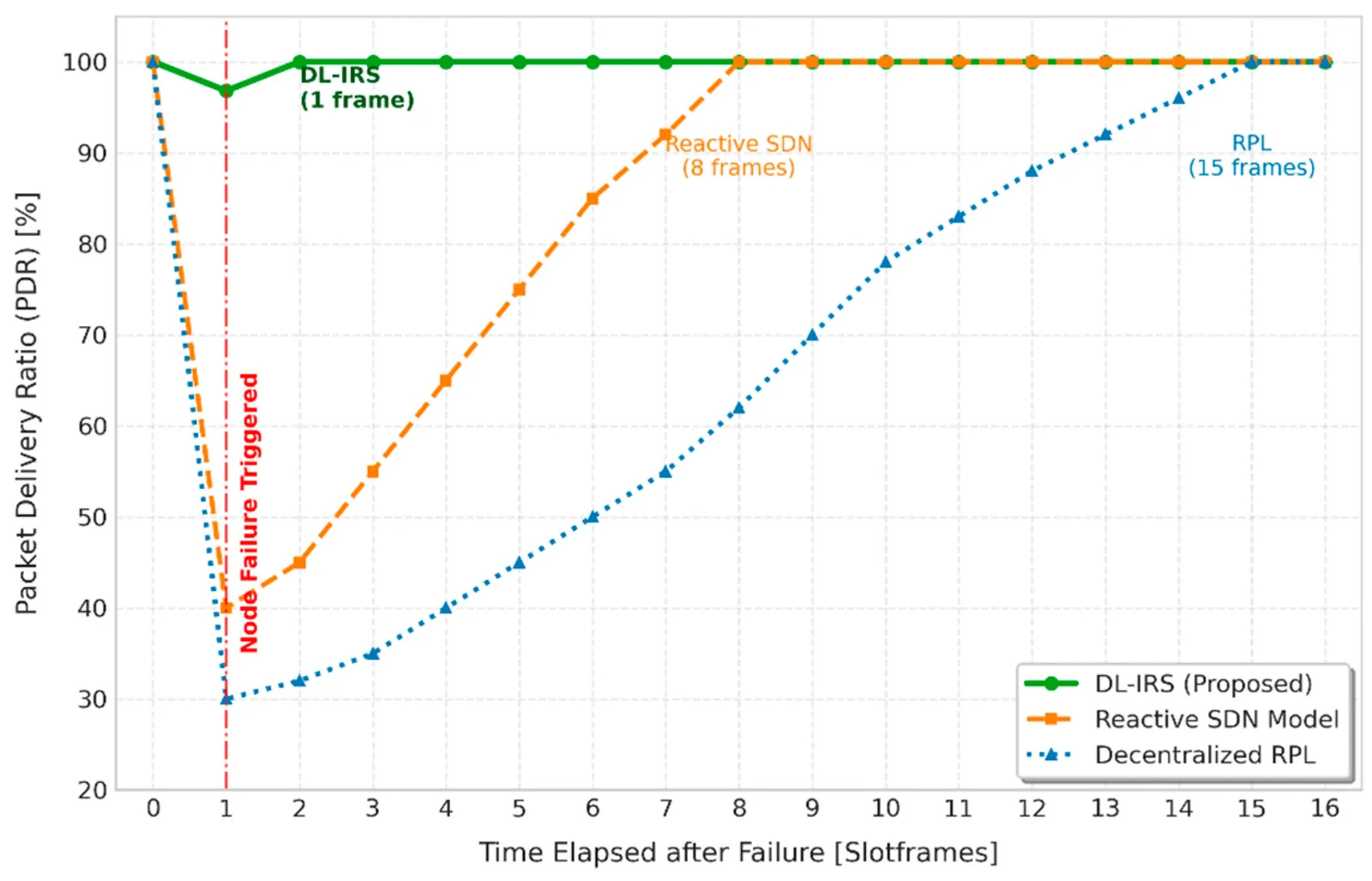
Stability-convergence time (PDR recovery) after a relay-node failure.

Following a critical relay-node failure, DL-IRS restores full PDR within one slotframe (Figure 5), compared with 8 slotframes for a reactive-SDN baseline and 15 slotframes for a decentralized RPL baseline under the same failure scenario. Figure 5 shows a small transient dip (to approximately 97%) at the failure instant rather than a literal zero-interruption transition.

**Figure 6:**
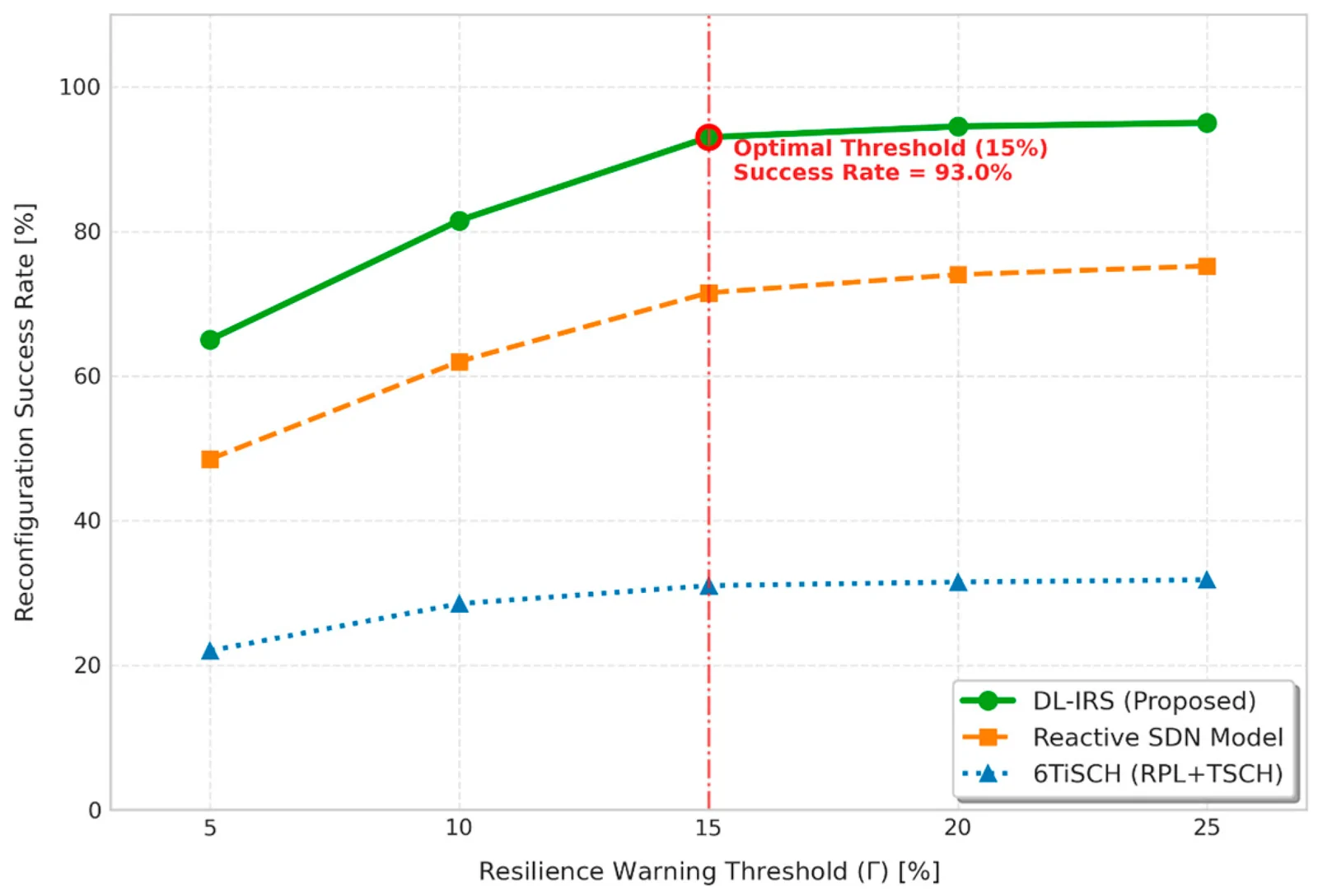
Reconfiguration success rate versus warning-threshold Γ, DL-IRS versus reactive-SDN and standard 6TiSCH.

**Figure 7:**
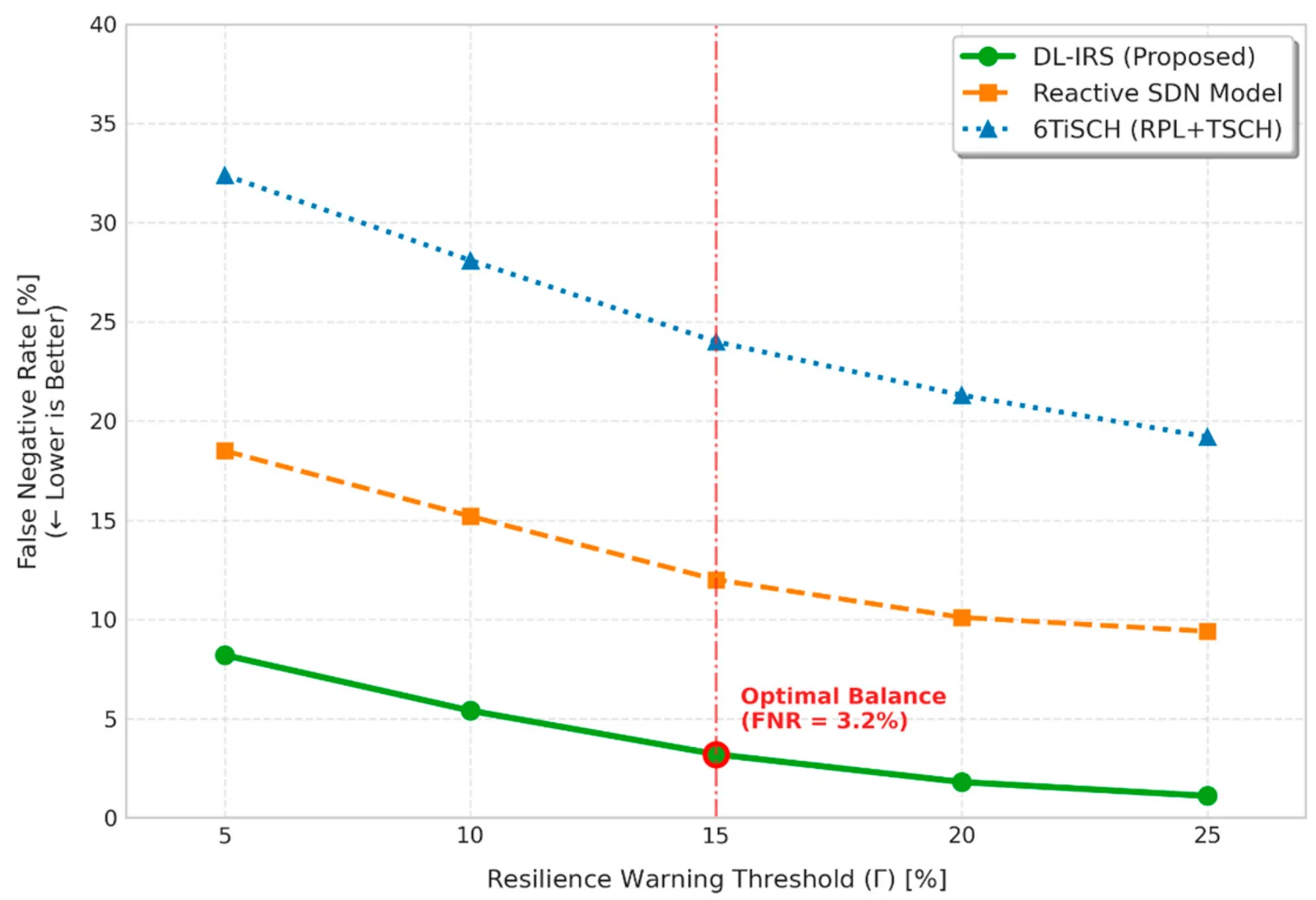
Trigger false-negative rate versus warning-threshold Γ, DL-IRS versus reactive-SDN and standard 6TiSCH.

Setting the warning-margin threshold *Γ_w_* to an absolute PRR margin of 0.05 yields a reconfiguration-success rate of 93.0% in preempting link outages at that operating point (Figure 6), with a corresponding false-negative rate of 3.2% (Figure 7).

### 4.3. Spatial Reuse and Scheduling Efficiency

**Figure 8:**
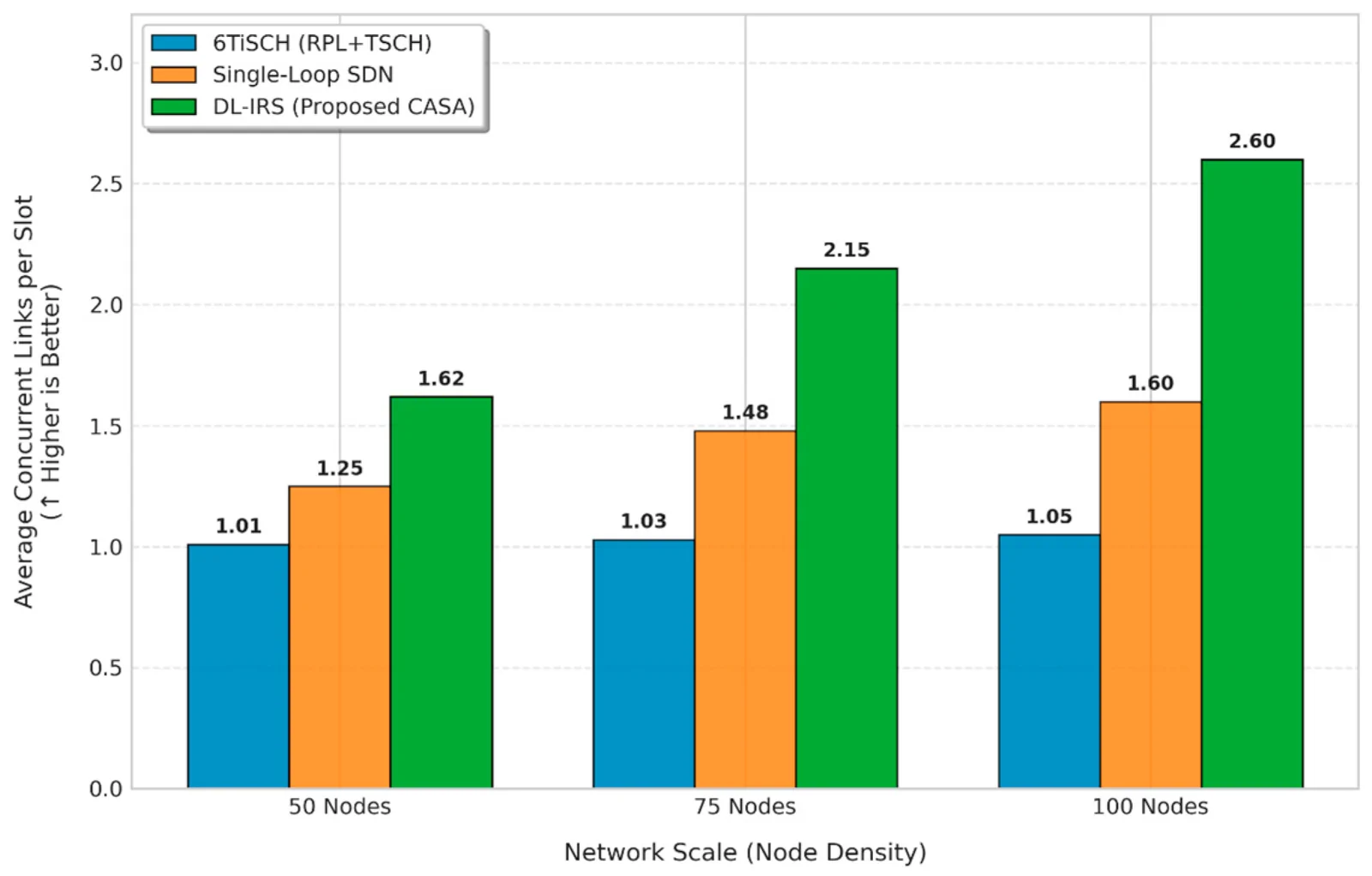
Average concurrent links per slot (ACLS), Equation (21), versus node density.

CASA (Section 3.4) mitigates bandwidth consumption through conflict-aware spatial multiplexing. Figure 8 reports ACLS (Equation (27)) across the 50/75/100-node topologies: DL-IRS sustains an average of 2.60 concurrent transmissions per slot at 100 nodes, versus 1.60 for the single-loop SDN baseline and 1.05 for standard 6TiSCH. In terms of normalized cell-occupancy ratio (SSRE, Equation (28)), DL-IRS achieves an absolute SSRE of 0.163 at 100 nodes compared to 0.066 for standard 6TiSCH, representing a relative SSRE gain (${SSRE}_{gain}$, Equation (29)) of +147.6%. Because SSRE = ACLS/C with C = 16 channel offsets (Section 3.4.2), these values follow directly from the ACLS values above (2.60/16 = 0.163 and 1.05/16 = 0.066); the single-loop SDN baseline reaches 0.100. The +147.6% figure is a relative gain, and shadow reservations are not counted because they are idle until activation.

### 4.4. Control Overhead, Energy, and Scalability

**Figure 9:**
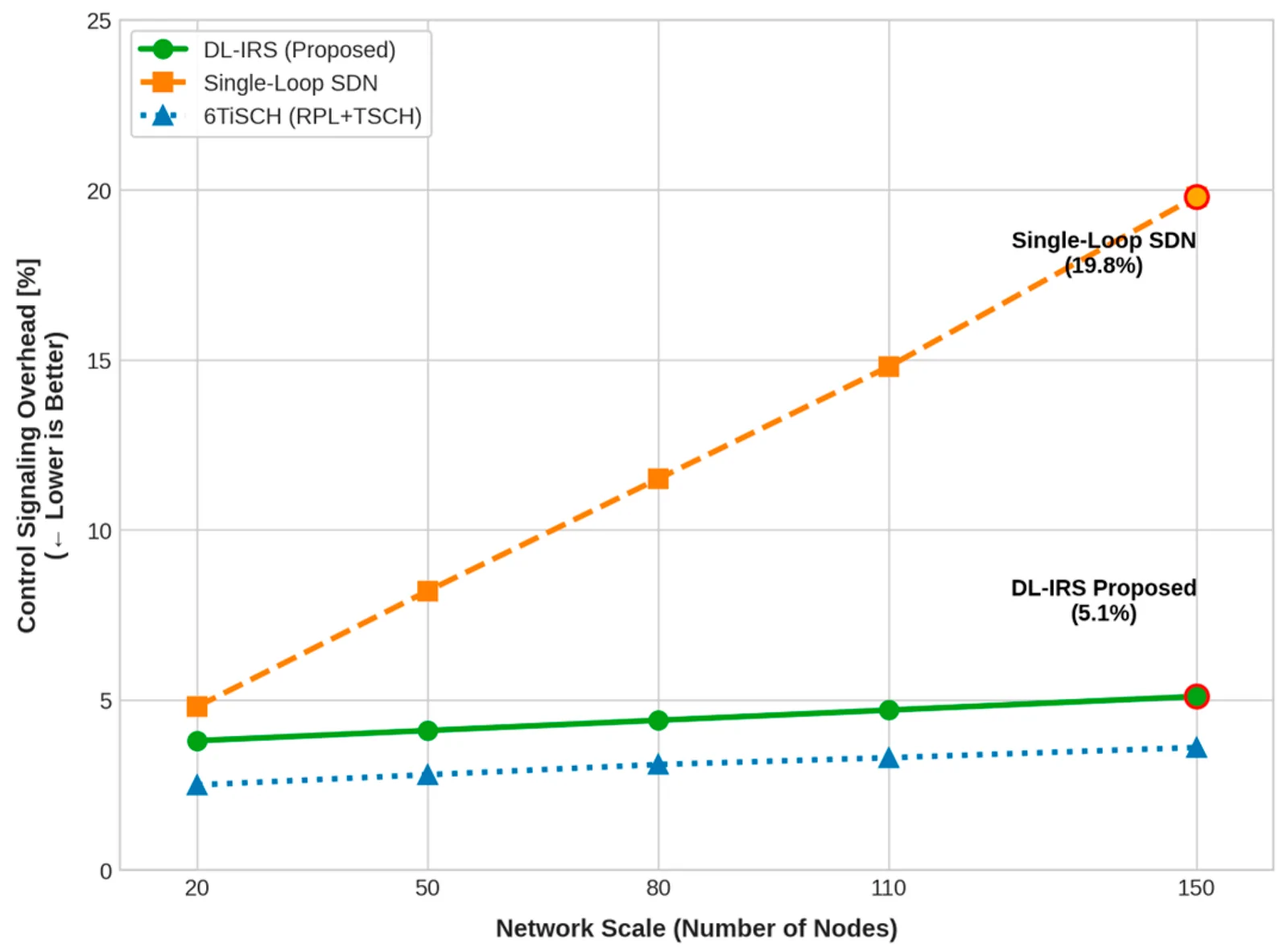
Control signaling overhead (% airtime) versus node count, 20–150 nodes.

**Figure 10:**
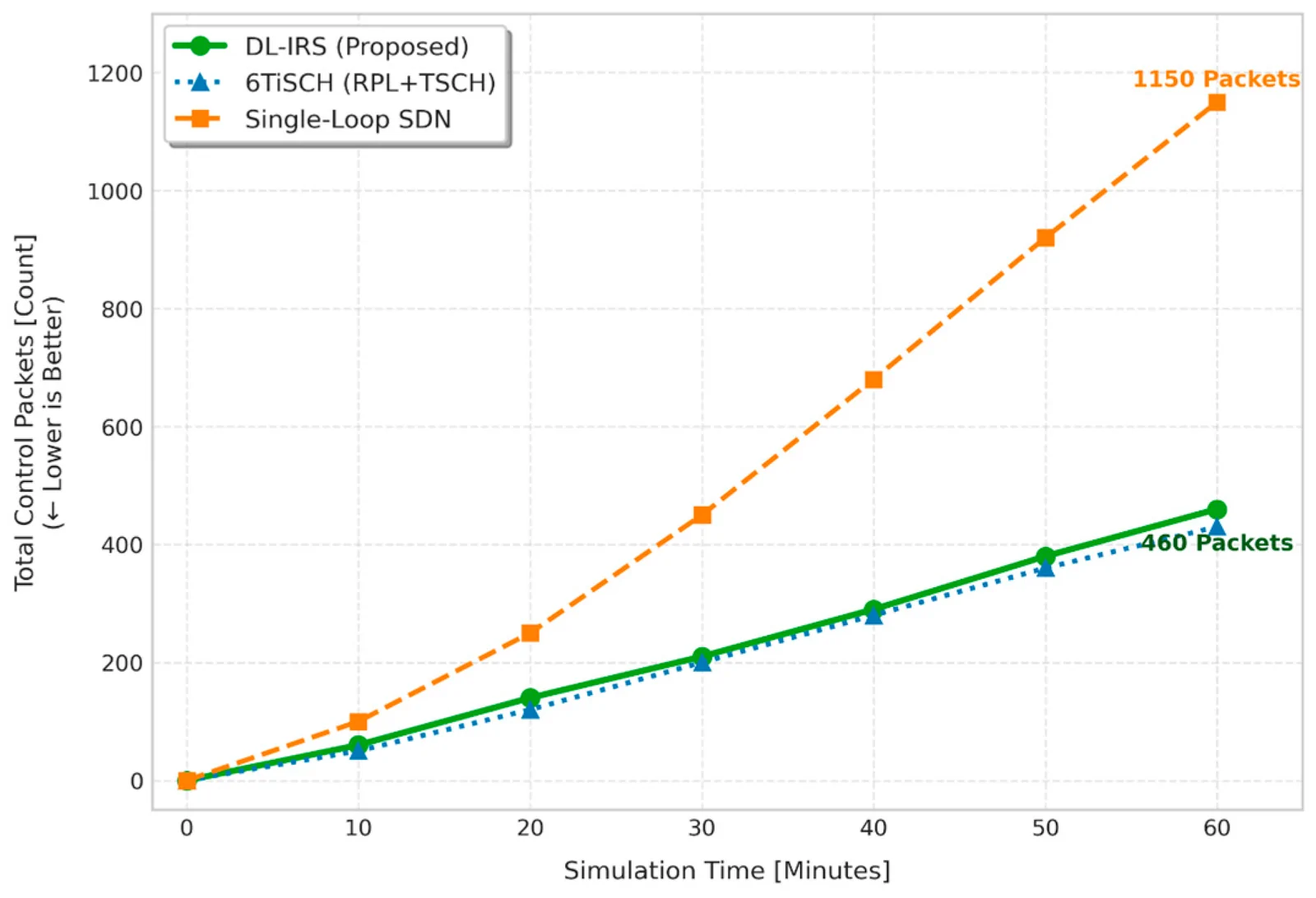
Total control packets versus simulation time, DL-IRS versus single-loop SDN and standard 6TiSCH.

As node density increases from 20 to 150 nodes, DL-IRS signaling overhead rises only from 3.8% to 5.1% of available airtime (Figure 9), while a single-loop SDN baseline rises to 19.8% overhead under the same sweep. Figure 10 reports the same qualitative behavior in absolute control-packet counts over a 60 min simulation window (460 packets for DL-IRS and standard 6TiSCH versus 1150 for single-loop SDN).

**Figure 11:**
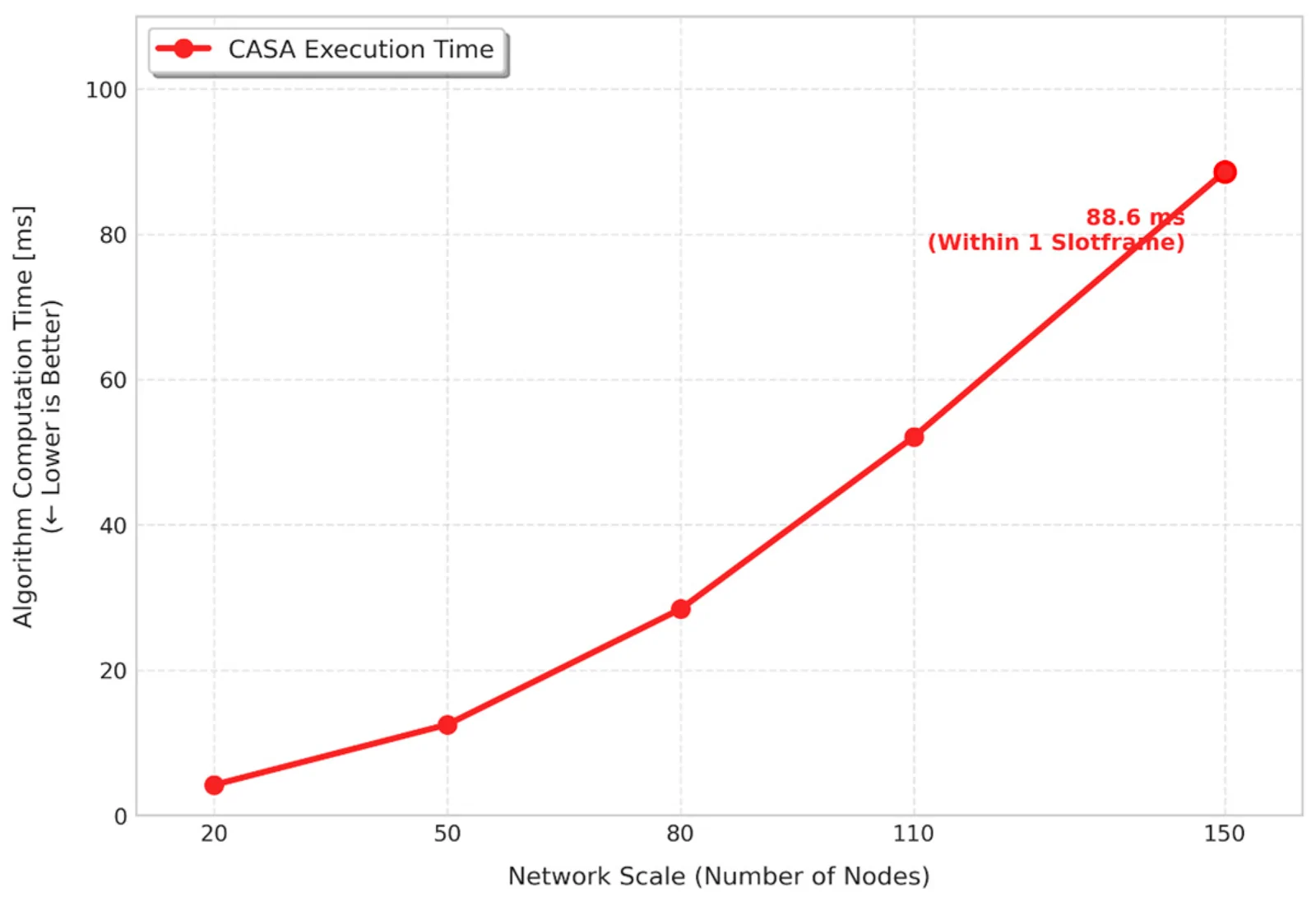
CASA controller computation time versus network scale.

Figure 11 reports CASA’s own controller-side computation time as a function of network scale: 88.6 ms at 150 nodes, which the figure annotates as remaining within one slotframe interval at the tested slotframe duration.

**Figure 12:**
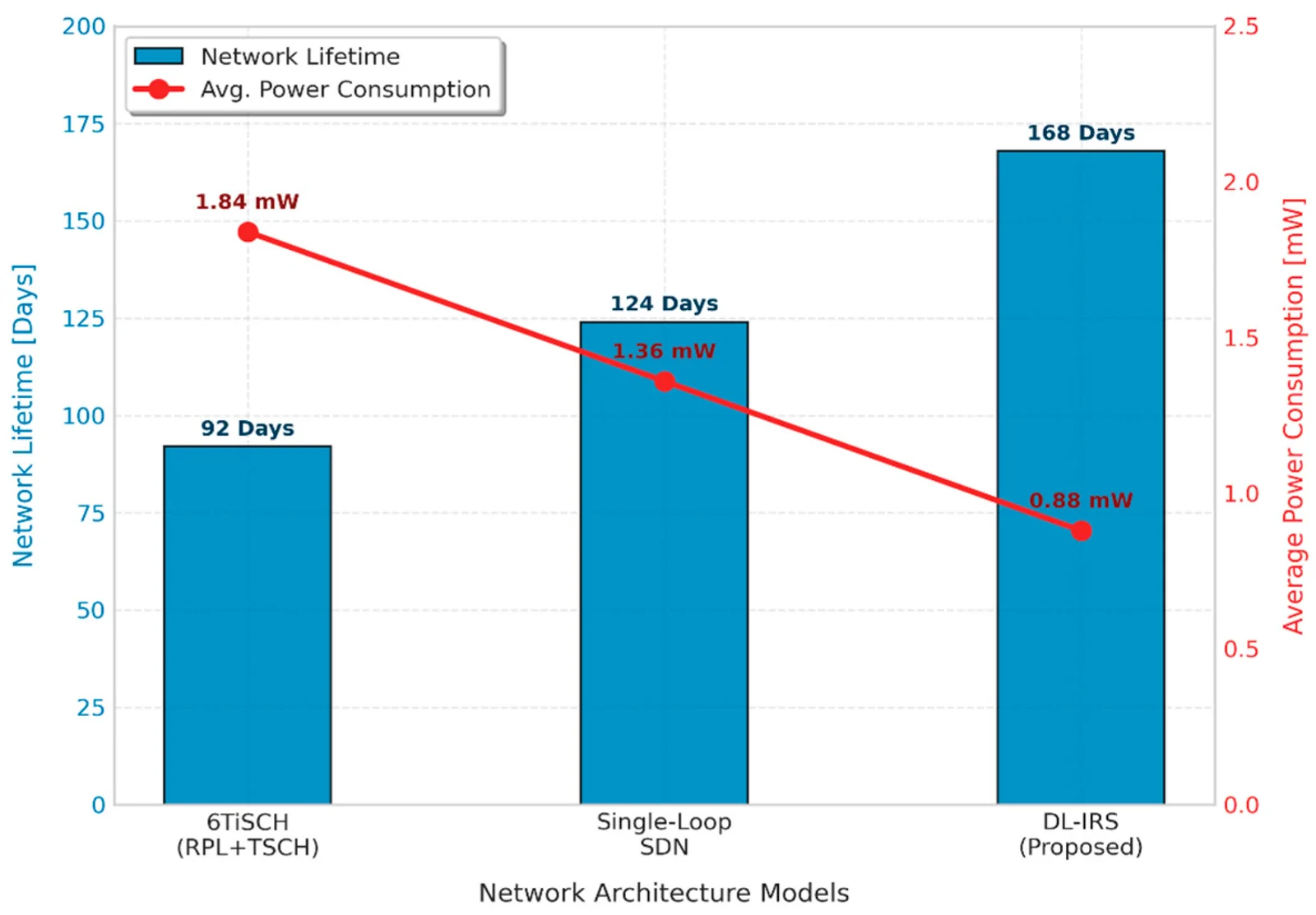
Network lifetime (First Node Death) and average power consumption, DL-IRS versus single-loop SDN and standard 6TiSCH.

Figure 12 reports network lifetime (First Node Death, FND) together with average control-plane power consumption across three architectures: standard 6TiSCH (92 days, 1.84 mW), single-loop SDN (124 days, 1.36 mW), and DL-IRS (168 days, 0.88 mW). Expressed in terms of energy per useful bit delivered at nominal throughput, DL-IRS consumes 0.95 μJ versus 1.98 μJ for standard 6TiSCH.

### 4.5. Optimality-Gap Study on Small Instances

To validate the solution quality of the CASA heuristic, we evaluated small-scale network instances (N∈{10, 15, 20, 25, 30} nodes) against an exact MILP oracle implemented in Gurobi 10.0. The oracle was configured with a time limit of 3600 s per instance. Table 8 summarizes the comparative objective values, optimality gaps ($100\times \frac{\Phi_{CASA}-\Phi_{MILP}}{\Phi_{MILP}}$), and execution runtimes.

### 4.6. Ablation and Sensitivity Analysis

**Figure 13:**
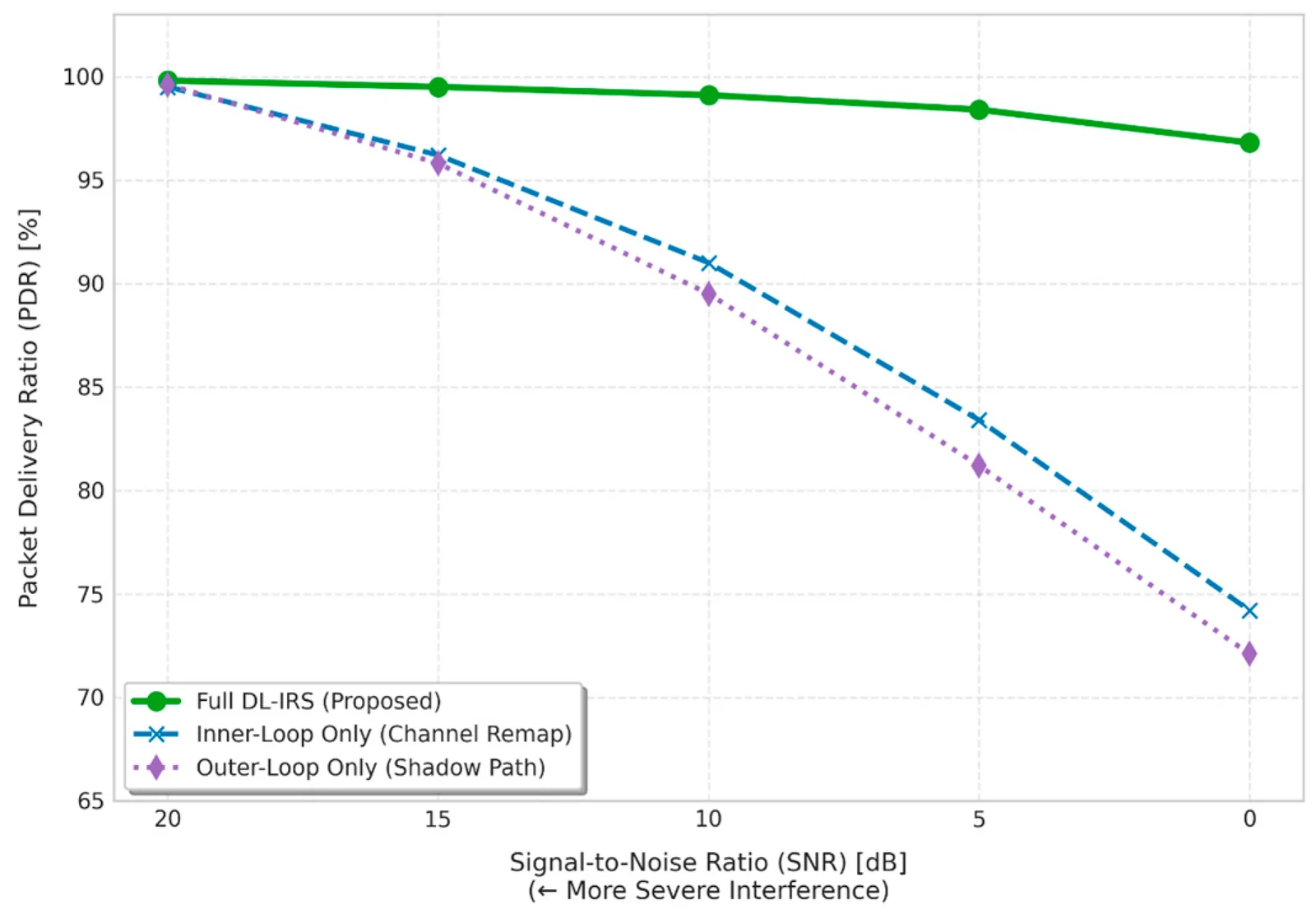
Ablation study: packet delivery ratio versus SNR for Full DL-IRS, Inner-Loop-Only, and Outer-Loop-Only variants.

Ablation analysis: Figure 13 reports an ablation comparison: Full DL-IRS versus Inner-Loop-Only versus Outer-Loop-Only variants across the SNR sweep. At SNR = 0 dB, Full DL-IRS sustains 96.8% PDR versus 74.2% for Inner-Loop-Only and 72.1% for Outer-Loop-Only. Table 9 extends the analysis to include a No-CASA variant at SNR = 0 dB.

#### 4.6.1. Statistical Protocol and Experiment Matrix

For every point in all figures, simulations were conducted across 20 independent random seeds. Reported error bounds represent 95% confidence intervals derived via Student-t distribution. Pairwise statistical significance was confirmed using the Mann–Whitney U test (p<0.01) with Cliff’s delta (d>0.82). The overall experimental evaluation design and controlled parameters are summarized in Table 10, while the precise mathematical metrics used to evaluate each performance claim are defined in Table 11. At the SNR = 0 dB operating point, all pairwise comparisons between DL-IRS and the baseline models yielded U=400.0 (the maximum possible rank sum for $n_{1}=n_{2}=20$), p<0.01, and Cliff’s δ=1.00, confirming strong statistical significance beyond the pre-specified thresholds. 

Table 12 provides the detailed statistical test results and metrics comparing DL-IRS against standard 6TiSCH and single-loop SDN at the SNR = 0 dB operating point.

#### 4.6.2. Parameter Sensitivity and Weight Selection

To show how the main design parameters influence performance, experiment E7 covers the trigger parameters (λ, $\Gamma_{w}$, K) and experiment E9 covers the objective weights (α,β,γ,η) of Equation (3) and the CASA weights ($\omega_{1},\ldots ,\omega_{5}$) of Equation (20). The trigger parameters are swept over the ranges in Table 13, and each weight is perturbed by ±50% with the remaining weights renormalized to unit sum, so that every configuration differs from the default in a single factor. All configurations use the 100-node topology at SNR = 0 dB with 20 seeds per point and the confidence-interval protocol of Section 4.6.1.

The sweep does not assert a single operating point as universally optimal: the defaults in Table 13 are the values used throughout Section 4, and Table 13 reports how far the PDR and the control overhead move when each factor is varied.

#### 4.6.3. Comparison with HPS and HRL-TSCH

Single-loop SDN is the primary simulation baseline because it shares the resource model, the controller substrate, and CASA with DL-IRS and lacks only the separation between local and global actions. The PDR, latency, and overhead differences of Section 4.2, Section 4.3 and Section 4.4 can therefore be attributed to the dual-loop structure, and the No-CASA variant of Table 9 isolates the allocator. HPS [17] and HRL-TSCH [20] differ from DL-IRS in several dimensions at once (Table 7), and a numerical comparison would additionally depend on how faithfully each is reproduced, for HRL-TSCH including its training episodes, convergence criteria, and inference overhead.

HPS and HRL-TSCH were not re-implemented in this study. The reported gains are therefore gains over standard 6TiSCH, reactive SDN, and single-loop SDN, and no numerical superiority over HPS or HRL-TSCH is claimed. Implementing both schedulers on the same Cooja topology, channel set, and traffic (Table 6) is the first quantitative extension identified in the Conclusion.

### 4.7. Discussion, Standards Mapping, and Limitations

#### 4.7.1. Standards-Aligned Positioning

DL-IRS is designed as an SDN-assisted scheduling layer above the TSCH data plane and is conceptually compatible with deterministic-networking principles: explicit resource reservation, bounded-latency forwarding, redundancy, and managed reconfiguration. The active and shadow schedules can be represented as controller-maintained forwarding and cell-allocation state, while the activation ASN provides a time-synchronized commit point. This work does not modify the IEEE 802.15.4 TSCH hopping operation and does not assert compliance with a specific IETF control-plane protocol. Instead, it identifies a standards-aligned implementation path in which schedule installation and telemetry can be conveyed through 6TiSCH management mechanisms, subject to message-format and interoperability validation in future work. Reliable and Available Wireless (RAW) and DetNet concepts are cited here as architectural context for bounded-latency, redundant forwarding.

#### 4.7.2. Simulation-Only Limitation

The evaluation reported in Section 4 is simulation-based and therefore cannot establish performance under all hardware-specific effects, including oscillator drift, radio-driver timing variance, firmware queue behavior, adjacent-channel leakage, and industrial non-line-of-sight fading. To mitigate this limitation, we disclose the simulator version, radio model, propagation/interference parameters, traffic-generation process, and random seeds in Table 5, and we test sensitivity to multiple interference conditions in experiment E6 (Section 4.6). The simulation includes a fading extension of the UDGM and multiple interference sources (Table 5), but it remains an abstraction of real radios. Two complementary validation steps will therefore be carried out before deployment claims are made: (i) a trace-driven experiment, in which per-channel PRR and RSSI traces recorded on a physical IEEE 802.15.4-TSCH deployment are replayed through Cooja to test the trigger logic of Section 3.4.1 against measured rather than synthetic interference dynamics; and (ii) a small hardware testbed of OpenWSN/CC2650-class nodes (for example, 10–20 nodes with a controllable narrowband interferer) in which the PDR, the recovery time, and the 38.0 ms detection-to-activation chain of Figure 2 are measured with the metric definitions of Table 11. Until these experiments are completed, the reported results should be read as simulation-based evidence only.

#### 4.7.3. Modeling Assumptions and Claim Scopes

Two modeling choices in Section 3 constrain the scope of the claims: first, the reliability linearization of Equation (2) assumes link-level reliability outcomes are conditionally independent given the schedule. Second, the shadow-schedule reservation semantics adopted for the reported results are dedicated (idle-until-activation) rather than preemptible. Furthermore, the outer-loop handover is not described as zero-delay. The term minimally disruptive data-plane handover is used instead: the switch at the activation ASN incurs no route recomputation (Section 3.4.4), while the 38.0 ms detection-to-activation chain (Figure 2) and the transient PDR dip (Figure 5) are reported explicitly. Similarly, the configured deadline is satisfied in 99.2% of simulated critical-flow packets under the evaluated conditions, absent a formal schedulability proof. Finally, the reported experiments exercise only the compute-on-trigger branch of Algorithm 3 (step 2); the proactive reservation of Phase A, and hence the claim that make-before-break removes the path search from the recovery chain, is a design property that has not been evaluated experimentally.

#### 4.7.4. Robustness Under Changing and Unseen Conditions

The evaluation covers the interference conditions of Table 5, whereas an industrial plant also exhibits operating conditions that change and that are not seen at design time. Recent work on machinery fault diagnosis, although developed for a different application domain, addresses the same structural problem: maintaining performance when the data distribution shifts to conditions absent from training. Prompt-guided disentanglement and fusion for cross-domain diagnosis with class mismatch [24], category-invariant disentangled features for domain generalization [25], and adaptive relation networks for settings with zero fault samples and fluctuating operating conditions [26] share the idea of separating condition-specific factors from the factors that determine the quantity of interest. By analogy, the EWMA-and-threshold monitor of DL-IRS is tuned to the interference models of Table 5 and may need re-tuning under unseen interference patterns; a learned link-quality estimator that disentangles interference-specific from link-quality-relevant features is one possible extension. Such an extension is not evaluated here, and the explicit, auditable optimization formulation remains the basis against which any learned component should be validated.

## 5. Conclusions

This paper formulated resilient scheduling in interference-prone 6TiSCH industrial wireless networks as an explicit mixed-integer linear program and proved that the underlying decision problem is NP-complete via a reduction from graph coloring (Proposition 1). Building on this formulation, we presented Dual-Loop Intelligent Resilient Scheduling (DL-IRS), which separates recovery into a local, minimum-perturbation inner loop and a predictive, make-before-break outer loop operating over a shared conflict-aware resource model (Table 1). We specified the Conflict-Aware Spatial-Spectral Allocation (CASA) heuristic independently of the space–time conflict graph it consumes, gave its conflict construction, scoring function, and pseudocode (Section 3.4.2), and positioned it as an empirically evaluated approximation to the NP-hard optimum.

In Cooja/Contiki-NG simulation, DL-IRS sustains a packet delivery ratio of 96.8% under an SNR = 0 dB narrowband-interference condition, an absolute improvement of 35.3 percentage points over standard 6TiSCH scheduling (61.5%) under matched topology and traffic, while reducing end-to-end latency at that same operating point from 78.4 ms (single-loop SDN) to 22.4 ms and constraining control-plane signaling overhead to 3.8–5.1% of airtime across a 20–150 node range. Estimated network lifetime (First Node Death) extends to 168 days under the tested energy model, compared with 124 days for a single-loop SDN baseline and 92 days for standard 6TiSCH. The recovery-timing results correspond to the compute-on-trigger branch of the outer loop; the Phase A proactive reservation is specified but remains to be evaluated.

Future work includes: (i) trace-driven and hardware validation on a physical IEEE 802.15.4-TSCH testbed to assess simulation-only limitations; (ii) extending the single-slotframe MILP formulation of Section 3.2 to multi-slotframe hyperperiod scheduling; (iii) investigating high-speed mobility scenarios, such as autonomous mobile robots; (iv) exploring a multi-agent reinforcement-learning variant of the CASA allocation policy; (v) cross-layer integration with energy-harvesting-aware scheduling; and (vi) quantitative comparison with re-implemented HPS and HRL-TSCH schedulers. In addition, a controlled evaluation of the Phase A proactive reservation (reserved-standby-valid versus compute-on-trigger cases, with Phase A enabled and disabled) is needed to validate the make-before-break claim.

## Figures and Tables

**Table 1:** Structural distinction between the inner and outer resilience loops.

Dimension	Inner Loop	Outer Loop
Trigger	Transient degradation on an active link/channel	Persistent degradation, node failure, or exhausted local alternatives
Decision Variables	Channel offset and activation ASN	Backup path, cells, shadow schedule, activation ASN
Scope	Affected link or local conflict neighborhood	End-to-end critical-flow path set
Scheduling Principle	Fixed-time, variable-frequency	Make-before-break, advance-reserved resource activation
Objective Priority	Minimum perturbation and low control overhead	Deadline preservation and structural resilience
Controller Action	Incremental channel-remap update	Shadow-schedule dissemination and atomic activation
Fallback	Escalate to outer loop if local recovery fails	Recompute a new standby schedule if existing backup is invalid

**Table 2:** Positioning of DL-IRS relative to representative prior scheduling mechanisms.

Mechanism	Recovery Trigger	Local/Global Scope Separation	Formal Resource Model
Standard 6TiSCH/RPL	Reactive (post-failure)	No—single recovery path	Protocol-defined, not optimization-formulated
HPS [17]	Proactive channel selection	No—single adaptation scope	Heuristic, no MILP reference
HRL-TSCH [20]	Learned policy	No—single learned action space	Implicit (reward-defined)
Single-loop SDN scheduling [23]	Centralized recomputation	No—one reconfiguration scope	Partial (architecture only)
DL-IRS (this work)	Local trend/threshold and global escalation	Yes—explicit inner/outer scope separation (Table 1)	Explicit MILP + NP-hardness result (Section 3)

**Table 3:** Notation used in the resilient scheduling MILP.

Symbol	Definition
G=(V, E)	Directed communication graph
F	Set of flows
$Q_{f}$	Route-copy indices of flow f
T,C	Slot offsets and channel offsets
$a_{f,\;e}$	1 if link e is selected for flow f; 0 otherwise
$z_{f,\;q,\;e}$	1 if link e belongs to copy q of flow f (decision)
$x_{f,\;q,\;e,\;t,\;c}$	1 if copy q of flow f uses link e in cell (t, c) (decision)
$y_{e,\;t,\;c}$	1 if any flow uses link e in cell (t, c) (auxiliary)
$b_{f,\;q,\;e,\;t,\;c}$	1 if cell (t, c) is reserved as a standby resource for protected copy q
$\chi_{e,\;e^{\prime }}$	1 if links e and $e^{\prime }$ cannot share a cell
$p_{e,\;c}$	Predicted PRR of link e at channel offset c
$l_{e,\;c}$	Reliability penalty, $-\log p_{e,\;c}$
$\varepsilon_{e,\;c}$	Transmission energy cost
$\delta_{t,\;t^{\prime }}$	Slot-distance contribution for precedence/delay

**Table 4:** Complexity of DL-IRS controller stages.

Stage	Worst Case	Typical Case	Notes
Path computation per flow	O(mlogn)	O(mlogn)	Standard shortest/disjoint-path routine
Conflict-graph construction	$O\left(m^{2}\right)$	$O\left(m^{2}\right)$	Pairwise link checks; reducible with neighborhood indexing
CASA allocation	O(F·H·T·C·(m+R))	$O\left(m^{2}+T\cdot C\right)$	Candidate check scans conflicting links; bounded repair depth R
Inner-loop remapping	$O\left(C\cdot d_{c}\left(e\right)\right)$	$O\left(C+d_{c}\left(e\right)\right)$	$d_{c}\left(e\right)$ is conflict-graph degree of affected link
Outer-loop rescheduling	$O\left(F_{c}\left[m\log n+H\cdot T\cdot C\cdot \left(m+R\right)\right]\right)$	$O\left(m^{2}+T\cdot C\right)$	$F_{c}$ is the affected critical-flow set
MILP oracle	Exponential worst case	Solver-dependent	Used only for small instances (Section 4.5)

**Table 5:** Reproducibility configuration parameters.

Category	Parameter/Requirement	Configured Value/Specification
Software	Operating System/Simulator	Contiki-NG v4.7/Cooja Simulator
	Compiler/Seed Policy	GCC 10.3/Random Seeds (20 independent runs per point)
Hardware Model	Mote Type/Radio	Zolertia Z1/CC2420 IEEE 802.15.4
	TX Power/RX Sensitivity	0 dBm/−95 dBm
TSCH Config	Slotframe Length/Slot Duration	101 slots/10 ms
	Hopping Sequence/Retries	16 Channels (2.4 GHz)/Max 3 Retries
Topology	Dimensions/Node Placement	100 m × 100 m/Grid with Random Jitter
	Node Counts	50, 75, 100 nodes (Density sweep: 20–150 nodes)
Traffic	Flow Types/Generator	Critical (Periodic, 100 ms)/Standard (Poisson)
Interference	NBI Models	WiFi Overlap (Ch 1, 6, 11) + Periodic EMI Pulses
Medium Model	Medium Propagation Model	Unit Disk Graph Model (UDGM) with Fading Extensions
Energy	Profiling Framework	Energest Module/Powertrace
Controller	Monitoring Parameters	EWMA λ=0.3, $\Gamma_{w}=0.05$, K=3 epochs
Scheduler Weights	MILP (α, β, γ, η)/CASA (ω1–ω5)	(0.4, 0.3, 0.2, 0.1)/(0.4, 0.2, 0.2, 0.1, 0.1)
Spatial Reuse	Channel offsets C/slotframe length T	C = 16, T = 101 (SSRE = ACLS/C)

**Table 9:** Comprehensive ablation matrix across key performance dimensions at SNR = 0 dB.

Variant	PDR (%)	p99 Delay (ms)	Deadline Miss Ratio (%)	Recovery Time (Frames)	Control Airtime (%)	Energy/Bit (μJ/bit)
Full DL-IRS	96.8%	28.4	0.8%	1.0	5.1%	0.95
Inner-Loop Only	74.2%	84.2	14.2%	N/A (Path Uncorrected)	3.9%	1.42
Outer-Loop Only	72.1%	92.6	18.5%	1.0	14.8%	1.85
No-CASA Allocator	81.5%	54.1	8.6%	2.4	5.2%	1.31

**Table 10:** Evaluation experiment matrix.

Experiment	Controlled Factor	Primary Output	What it Proves
E1 Reliability under NBI	SNR/interference duty cycle/channel overlap	PDR by traffic class, deadline-miss ratio	Robustness under controlled interference
E2 Failure recovery	Relay-failure time and location	Detection/install/activation/recovery breakdown	Detection-to-activation chain of the compute-on-trigger branch (the Phase A reservation-hit path is not evaluated)
E3 Density scaling	20–150 nodes	Controller runtime, feasibility, control airtime, PDR	Scalability claim
E4 Load scaling	Offered load and critical-flow proportion	PDR, p99 delay, queue drops	Behavior under bursty traffic
E5 Optimality gap	Small topologies (10–30 nodes)	MILP gap, runtime, feasibility	Technical quality of the CASA heuristic
E6 Interference-model sensitivity	≥2 documented radio/channel models	Outcome robustness	Whether results depend on one idealized model
E7 Threshold sensitivity	λ, $\Gamma_{w}$, K	FPR, FNR, recovery success, update rate	Justifies the selected trigger operating point
E8 Ablation	Full/no-inner/no-outer/no-CASA	All key metrics in common scenarios	Causal attribution of each design component
E9 Weight sensitivity	α, β, γ, η and ω1–ω5 perturbed by ±50% (one factor at a time)	PDR, p99 delay, update rate	Whether the schedule quality depends on the selected weights

**Table 11:** Metric definitions used for the corresponding claims.

Claim	Metric Definition
Reliability	Packet delivery ratio; duplicate-delivery success for protected flows; 95% confidence interval
Determinism	Mean, median, p95, p99, and maximum end-to-end delay; deadline-miss ratio; jitter definition
Recovery	Detection delay, computation delay, dissemination/install delay, activation delay, recovery time, packets lost during transition
Spatial reuse	ACLS (Equation (21)) and normalized SSRE (Equation (22)) or SSRE gain (Equation (23)), with unambiguous denominators
Control cost	Control bytes, control packets, airtime, and percentage of total airtime; periodic monitoring reported separately from reconfiguration updates
Energy	Energy per delivered useful bit and node lifetime; includes control energy and retransmission energy
Scalability	Controller runtime, schedule-feasibility ratio, memory use if possible, overhead growth with node/flow counts
Optimality	Objective gap $100\times \frac{\Phi_{CASA}-\Phi_{MILP}}{\Phi_{MILP}}$for small feasible instances; runtime comparison

**Table 12:** Test statistics at the SNR = 0 dB operating point (20 seeds per arm).

Comparison	Metric	DL-IRS: Mean ± 95% CI	Baseline: Mean ± 95% CI	U	*p*	Cliff’s δ
DL-IRS vs. standard 6TiSCH	PDR (%)	96.8 ± 0.8%	61.5 ± 1.9%	400.0	<0.001	1.00
DL-IRS vs. single-loop SDN	PDR (%)	96.8 ± 0.8%	76.2 ± 1.4%	400.0	<0.001	1.00
DL-IRS vs. single-loop SDN	Latency (ms)	22.4 ± 1.1 ms	78.4 ± 3.2 ms	400.0	<0.001	1.00

**Table 13:** Parameter sensitivity design and observed effect at SNR = 0 dB.

Parameter	Default	Range Swept	Observed Effect
EWMA factor λ	0.3	{0.1, 0.2, 0.3, 0.5, 0.7}	[insert PDR, FNR, and update-rate range]
Warning threshold Γw	0.05 (absolute PRR margin)	See Figure 6 and Figure 7	The false-negative rate of DL-IRS decreases monotonically as the threshold is raised in Figure 6 (8.2% to 1.1% over the swept range)
Persistence K	3 epochs	{1, 2, 3, 5}	[insert PDR and escalation-rate range]
Objective weights (α, β, γ, η)	(0.4, 0.3, 0.2, 0.1)	Each ±50%, renormalized	[insert PDR and p99-delay range]
CASA weights (ω1–ω5)	(0.4, 0.2, 0.2, 0.1, 0.1)	Each ±50%, renormalized	[insert PDR and p99-delay range]

## Data Availability

The data presented in this study are available on request from the corresponding author.

## References

[1] (2020). IEEE Standard for Low-Rate Wireless Networks.

[2] Vilajosana X., Watteyne T., Chang T., Vučinić M., Duquennoy S., Thubert P. (2020). IETF 6TiSCH: A tutorial. IEEE Commun. Surv. Tutor..

[3] De Guglielmo D., Brienza S., Anastasi G. (2016). IEEE 802.15.4e: A survey. Comput Commun..

[4] Queiroz D.V., Alencar M.S., Gomes R.D., Fonseca I.E., Benavente-Peces C. (2017). Survey and systematic mapping of industrial Wireless Sensor Networks. J. Netw. Comput Appl..

[5] Balbi M., Doherty L., Watteyne T. (2025). A comprehensive survey on channel hopping and scheduling enhancements for TSCH networks. J. Netw. Comput Appl..

[6] Urke A.R., Kure Ø., Øvsthus K. (2022). A survey of 802.15.4 TSCH schedulers for a standardized industrial Internet of Things. Sensors.

[7] Ghanbari Z., Navimipour N.J., Hosseinzadeh M., Shakeri H., Darwesh A. (2022). The applications of the routing protocol for low-power and lossy networks (RPL) on the internet of mobile things. Int. J. Commun. Syst..

[8] Saifullah A., Xu Y., Lu C., Chen Y. Real-time scheduling for WirelessHART networks. Proceedings of the 2010 31st IEEE Real-Time Systems Symposium.

[9] Raut A.R., Khandait S., Theng D. (2024). A priority based self-organised MAC protocol for real time wireless sensor network applications. Eng. Technol. Appl. Sci. Res..

[10] Kalita A., Hazra A., Gurusamy M. Efficient schemes for improved performance in 6TiSCH networks. Proceedings of the IEEE INFOCOM 2023 Conference on Computer Communications Workshops (INFOCOM WKSHPS).

[11] Kalita A., Gurusamy M. (2024). On-the-fly autonomous slot allocation in 6TiSCH-based industrial IoT networks. IEEE Trans. Ind. Inf..

[12] Lee J.H., Chung S.H. (2025). Adaptive cell scheduling and negotiation techniques for 6TiSCH networks under bursty traffic. Sensors.

[13] Lee H., Chung S.H., Kim J.D. (2025). Cell scheduling for enhanced bidirectional throughput in TSCH networks. IEEE Access.

[14] Aimaretto L., Dujovne D. (2023). BDPC: Controlling application delay in 6TiSCH networks for the industrial Internet of Things. Internet Things.

[15] Aimaretto L., Dujovne D. (2023). Enhancing end-to-end determinism and reliability in 6TiSCH networks with disjoint leaf-based MPLS-like tunnels. Internet Things.

[16] Zhang Y., Xu S., Huang Q., Han Y. (2026). Kalman filter scheduling for 6TiSCH network with traffic adaptation optimized for bursty traffic. Ad. Hoc Netw..

[17] Tabouche A., Djamaa B., Senouci M.R. (2024). HPS: A hybrid proactive scheduler with adaptive channel selection for industrial 6TiSCH networks. Ad. Hoc Netw..

[18] Nunez D., Wilhelmi F., Galati-Giordano L., Geraci G., Bellalta B. Spatial reuse in IEEE 802.11bn coordinated multi-AP WLANs: A throughput analysis. Proceedings of the 2024 IEEE Conference on Standards for Communications and Networking (CSCN).

[19] Nunez D., Wilhelmi F., Galati-Giordano L., Geraci G., Bellalta B. (2026). Improving Wi-Fi 8 latency with coordinated spatial reuse. IEEE Commun. Stand Mag..

[20] Jurado-Lasso F.F., Orfanidis C., Jurado J.F., Fafoutis X. (2024). HRL-TSCH: A hierarchical reinforcement learning-based TSCH scheduler for IIoT. IEEE Trans. Cogn. Commun. Netw..

[21] Ben Yaala S., Ben Yaala S., Bouallegue R. (2025). Optimizing TSCH scheduling for IIoT networks using reinforcement learning. Technologies.

[22] Mtowe D.P., Kim D.M. (2023). Edge-computing-enabled low-latency communication for a wireless networked control system. Electronics.

[23] Balasubramanian V., Aloqaily M., Reisslein M. (2021). An SDN architecture for time sensitive industrial IoT. Comput Netw..

[24] Chen Z., Wu J., Huang H. (2026). Prompt-guided disentanglement and fusion framework for cross-domain fault diagnosis with class mismatch learning. IEEE Trans. Ind. Inf..

[25] Chen Z., Wu J., Deng Z., Huang H. (2026). Learning category-invariant disentangled features for domain generalization in machine fault diagnosis. IEEE/ASME Trans. Mechatron..

[26] Chen Z., Wu J., Deng Z., Huang H. (2026). Adaptive relation network for rotating machinery fault detection in zero fault samples and fluctuating operating conditions. IEEE Trans. Syst. Man. Cybern. Syst..

